# Nanoscale architecture of synaptic vesicles and scaffolding complexes revealed by cryo-electron tomography

**DOI:** 10.1073/pnas.2403136121

**Published:** 2024-06-26

**Authors:** Richard G. Held, Jiahao Liang, Axel T. Brunger

**Affiliations:** ^a^Department of Molecular and Cellular Physiology, Stanford University, Stanford, CA 94305; ^b^Department of Neurology and Neurological Sciences, Stanford University, Stanford, CA 94305; ^c^Department of Structural Biology, Stanford University, Stanford, CA 94305; ^d^Department of Photon Science, Stanford University, Stanford, CA 94305; ^e^HHMI, Stanford University, Stanford, CA 94305

**Keywords:** synapse, cryo-electron tomography, synaptic transmission, nanoscale topography

## Abstract

The spatial distribution of proteins and their arrangement within the cellular ultrastructure regulates the opening of α-amino-3-hydroxy-5-methyl-4-isoxazolepropionic acid (AMPA) receptors in response to glutamate release at the synapse. Fluorescence microscopy imaging revealed that the postsynaptic density (PSD) and scaffolding proteins in the presynaptic active zone (AZ) align across the synapse to form a trans-synaptic “nanocolumn,” but the relation to synaptic vesicle release sites is uncertain. Here, we employ focused-ion beam (FIB) milling and cryoelectron tomography to image synapses under near-native conditions. Improved image contrast, enabled by FIB milling, allows simultaneous visualization of supramolecular nanoclusters within the AZ and PSD and synaptic vesicles. Surprisingly, membrane-proximal synaptic vesicles, which fuse to release glutamate, are not preferentially aligned with AZ or PSD nanoclusters. These synaptic vesicles are linked to the membrane by peripheral protein densities, often consistent in size and shape with Munc13, as well as globular densities bridging the synaptic vesicle and plasma membrane, consistent with prefusion complexes of SNAREs, synaptotagmins, and complexin. Monte Carlo simulations of synaptic transmission events using biorealistic models guided by our tomograms predict that clustering AMPARs within PSD nanoclusters increases the variability of the postsynaptic response but not its average amplitude. Together, our data support a model in which synaptic strength is tuned at the level of single vesicles by the spatial relationship between scaffolding nanoclusters and single synaptic vesicle fusion sites.

## Significance

Imaging the ultrastructure and molecular architecture of synapses is essential to understanding synaptic neurotransmission. Scaffolding proteins on both sides of the synapse form subsynaptic clusters that are aligned across the synapse. This is thought to concentrate the proteins required for presynaptic vesicle fusion across from postsynaptic receptors to mediate efficient neurotransmission. We used focused-ion beam milling and cryoelectron tomography to obtain 3D images of synapses under near-native conditions, allowing visualization of both synaptic vesicles and clustered scaffolding proteins. While scaffolding complexes are aligned across the synapse, membrane-proximal synaptic vesicles are offset from clustered scaffolds, suggesting a role for these geometric properties of synapses in determining the amplitude and variability of the synaptic response to vesicle fusion.

Neuronal synapses are specialized signaling compartments responsible for information transfer in the nervous system. Glutamatergic synapses constitute the majority of synapses in the brain (1). During an action potential, presynaptic vesicles fuse with the plasma membrane, releasing the neurotransmitter glutamate, which diffuses across the synaptic cleft to bind ionotropic glutamate receptors, including AMPA and NMDA receptors. AMPA receptors carry most of the postsynaptic current in response to synaptic vesicle fusion events (2–5). They are present at a high density at the synapse and have a relatively low affinity for glutamate (6). As a result, fusion of a single synaptic vesicle does not saturate AMPARs (3, 7). Instead, AMPAR opening probability depends on the distance from the site of synaptic vesicle fusion, decaying over 50% within 40 nm (8–11). This dependence of glutamatergic synaptic transmission on AMPAR nanoscale topography suggests possible mechanisms for regulating synaptic signal transduction and synaptic plasticity.

The positioning of postsynaptic AMPA receptors and presynaptic vesicle fusion sites are controlled by the postsynaptic density (PSD), the presynaptic active zone (AZ), and trans-synaptic cell adhesion complexes in the synaptic cleft. The PSD is a multiprotein, intracellular scaffolding complex that abuts the postsynaptic plasma membrane (12). The core scaffold of the PSD, the abundantly expressed protein PSD-95, interacts with AMPAR auxiliary subunits to anchor AMPARs at the synapse (13, 14). Protein interactions within the PSD also indirectly link AMPARs to downstream signaling pathways (13, 15). In the presynapse, synaptic vesicle fusion is driven by the core fusion machinery consisting of SNARE proteins, which provide the energy for membrane fusion (16, 17), synaptotagmins, which trigger fusion upon Ca^2+^ binding (18, 19), and complexins, which regulate the process (20–24). The AZ acts upstream of the core fusion machinery and, like the PSD, consists of large, multidomain proteins, notably Munc13 and RIM (25–27). The AZ complex is precisely localized to the synapse and controls synaptic vesicle maturation to a fusion-competent state, a process known as priming (25, 28–30). Munc13 is essential for synaptic vesicle priming and proper assembly of fusogenic SNARE complexes. It requires other AZ proteins, particularly RIM, for proper localization and function (26, 31–34). Loss of Munc13, or disruption of the AZ, results in a loss of membrane-proximal synaptic vesicles, suggesting that these vesicles are the morphological correlates of functionally primed vesicles (29, 30, 33).

The PSD and AZ span several hundred nanometers in diameter within a single synapse. Superresolution fluorescence microscopy revealed that both the PSD and the AZ form subsynaptic clusters spanning tens of nanometers in diameter—i.e., nanoclusters (9, 10, 35–37). Within the PSD, nanoclusters of PSD-95 align with similar clusters of AMPARs, consistent with the scaffolding function of PSD-95 (9, 35). Within the AZ, the number of Munc13 nanoclusters correlates with functionally defined release sites, and nanoclusters of RIM spatially correlate with sites of vesicle fusion events (10, 36). Fusion events also occur in spatial clusters, suggesting AZ nanoclusters are molecular markers of synaptic vesicle fusion sites (38). Furthermore, AZ and PSD nanoclusters are preferentially aligned across the synapse in trans-synaptic “nanocolumns” (10, 39). These data led to the model that synapses maximize the opening probability of AMPARs during synaptic transmission by precisely aligning vesicle fusion to AMPAR clusters using nanocolumns (8–10, 35). However, these previous fluorescence microscopy studies did not reveal the topographical relationships between AZ and PSD nanoclusters to membrane-proximal synaptic vesicles since membranes are not visible in these experiments.

Cryoelectron tomography (cryo-ET) is an imaging method that enables the three-dimensional (3D) reconstruction of cellular volumes from samples in “near-native” conditions at nanometer resolutions, frozen in vitreous ice without exogenous contrast agents or fixatives (40). Cryo-ET enables imaging of high-resolution 3D cellular ultrastructure, protein morphology, and, in favorable cases, near atomic-resolution protein structures in situ by subtomogram averaging (41–43). Previously, cryo-ET was applied to neuronal synapses using purified synaptosomes: synapses that have been sheared away from the cell soma and isolated via centrifugation (44–47). These studies revealed the presence of protein “tethers” linking synaptic vesicles to the AZ plasma membrane, as well as intervesicular linkers connecting synaptic vesicles within the presynaptic terminal (44). Still, the resolution achieved was insufficient to assign specific proteins to these densities. Therefore, knock-out mice were used to reveal the molecular identity of these tethers (45, 47). As an alternative to synaptosomes, synapses formed by cultured neurons were directly imaged by cryo-ET using high microscope defocus (−10 to −18 μm) or a Volta phase-plate to improve image contrast and correlated light and electron microscopy (CLEM) to identify morphological markers of excitatory and inhibitory PSDs (48–50). A fundamental limit to these earlier studies was sample thickness relative to the incident electron beam. High energy (300 keV) electrons have a mean-free path for inelastic scattering between 300 and 400 nm (51, 52). Samples greater than this thickness, even small synapses in the range of 0.6 to 1 μm in diameter (53, 54), suffer from a loss of image contrast due to increased inelastic scattering events, contributing to noise and limiting image resolution (55, 56). Samples of synapses therefore require compression during sample blotting to achieve acceptable sample thickness for cryo-ET which may cause artifacts.

To overcome the limitation in achieving optimal sample thickness, focused-ion beam (FIB) milling can be used to thin cellular samples into lamellae with thicknesses more amenable to cryo-ET (~150 to 200 nm) (57–59). To achieve optimal imaging conditions for the study of synapses using cryo-ET, we used FIB milling of cultured hippocampal neurons, targeting synapses formed along dendrites. We could reliably capture synapses in FIB-milled lamellae by targeting dense fasciculated neurites for milling. Tomograms reconstructed from these lamellae samples showed dramatic improvements in image contrast and resolution compared to previous studies of synaptosomes and nonmilled neurons, revealing detailed synapse ultrastructure under near-native conditions. We imaged presynaptic protein densities and investigated trans-synaptic alignment between AZ and PSD nanoclusters and their relationship to membrane-proximal synaptic vesicles. Using our tomograms, we then conducted tomogram-guided Monte Carlo simulations of synaptic transmission to predict the functional implications of the observed synapse architectures.

## Results

### Focused Ion Beam Milling of Cultured Neurons Can Target Synapses.

**Fig. 1.**
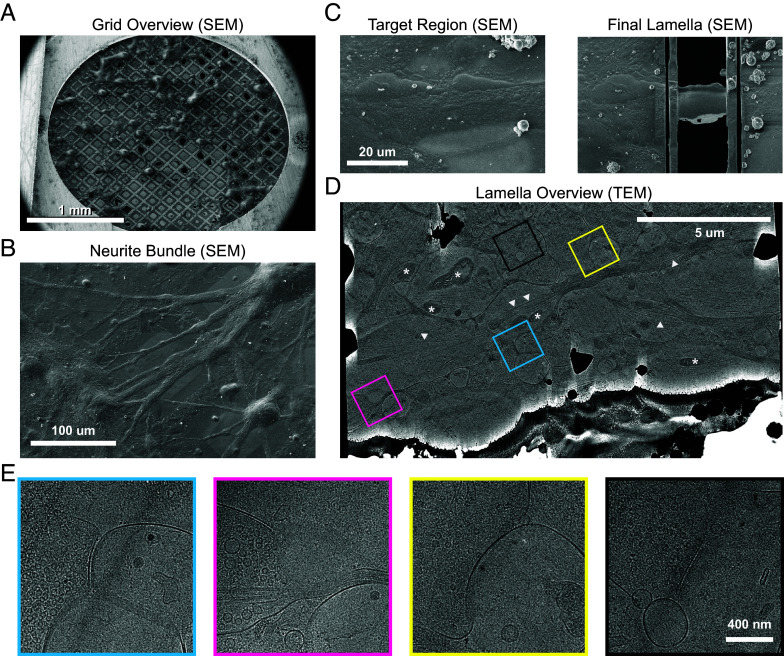
Cryo-FIB milling targeting neuronal synapses. (*A*) Cryo-SEM overview image of a vitrified culture of primary mouse hippocampal neurons grown on a holey carbon grid. (*B*) Cryo-SEM image of bundled neurites arborized from cell bodies. (*C*) Target selection for FIB milling shown from the SEM perspective. The *Left* panel shows a neurite bundle before milling and the *Right* panel shows the same region after polishing the final lamella. SEM images were acquired at 3 kV, 13 pA. (*D*) TEM search montage overview of the lamella shown in (*C*), at a pixel size of 3.83 nm/pixel and processed with the LisC algorithm (60). Colored boxes indicate the sites of putative synapses. White asterisks: mitochondria; white arrowheads: microtubules. (*E*) Search images (3.83 nm/pixel) of the boxed regions from (*D*). The presence of a synapse is indicated by presynaptic vesicles (*Left* side) opposite a relatively empty postsynaptic compartment (*Right*), separated by a synaptic cleft. Depending on the orientation of the synapse, the cleft is more or less apparent in these projection images.

To minimally perturb synapses before imaging, we imaged synapses from primary cultured hippocampal neurons grown directly onto holey carbon grids within a 35-mm culture dish. Hippocampal cultures were seeded at an intermediate density to favor abundant synapse formation while allowing for the removal of culture media by backside sample blotting during plunge freezing. Cryo-SEM imaging generally revealed large cell soma emanating branched arborizations (Fig. 1*A*). Closer inspection revealed that these branches consisted of bundles of neurites running mainly in parallel (Fig. 1*B*.) This phenomenon may be attributable to the absence of glial cells, which adhered poorly to the grids, or increased mechanical support provided by neurite bundling. Nevertheless, glial cells were visible by light microscopy on the glass layer of the culture dish. Neurite bundles provided tractable targets for sample thinning by focused ion beam (FIB) milling since they were larger than isolated synapses.

We targeted neurite bundles for FIB milling, reasoning that these bundles of axons and dendrites were likely to contain an abundance of synapses (Fig. 1*C*). After transfer to TEM, low-magnification lamellae overviews revealed neurites containing abundant microtubules, mitochondria, and synapses captured in cross-section (Fig. 1 *D* and *E*). Synapses were identified at low magnification by the presence of synaptic vesicles with a diameter of ~45 nm in presynaptic terminals opposed to rounded postsynaptic compartments. These postsynaptic compartments are presumably dendritic spine heads, as they do not contain microtubules, which are restricted mainly to the dendritic shaft and axons (54). Depending on the orientation of the synapse to the plane of the lamella, the synaptic cleft was often visible as an increased intermembrane distance between pre- and postsynaptic compartments (54). We targeted these putative synapses for tomographic tilt-series data collection and confirmed the presence of a synapse in the reconstructed tomogram.

### Cryo-ET of FIB-Milled Synapses Reveals High-Contrast Ultrastructure.

Reconstructed synapse tomograms from FIB-milled samples showed dramatic improvements in image quality and sample contrast compared to many previous studies (44–49). This is exemplified by the ability to clearly resolve leaflets of lipid bilayers and accurately fit contrast-transfer functions across a range of defocus values (*SI Appendix*, Fig. S1). The quality of our tomograms is comparable to that of isolated synaptic vesicle preparations (61), thus representing the maximum achievable quality with current cryo-ET technology.

**Fig. 2.**
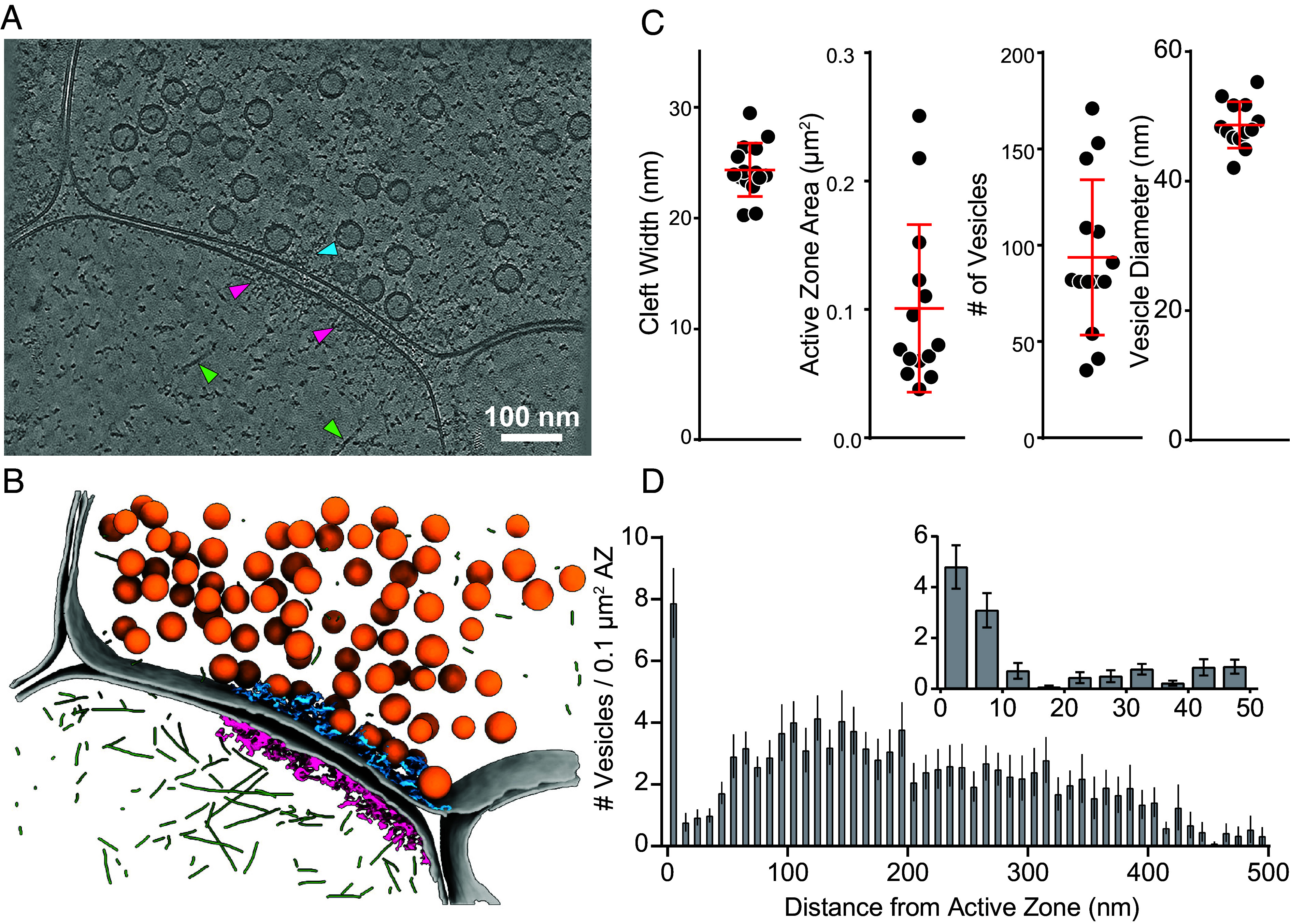
Ultrastructure analysis of FIB-milled synapse tomograms. (*A*) A single XY section through a tomogram of a synapse, reconstructed at bin 8 (1.36 nm/pixel) and denoised with cryoCARE (62). Cyan arrowhead: presynaptic protein density; Magenta arrowheads: PSD protein density; Green arrowheads: actin filaments. (*B*) Segmentation of the tomogram in (*A*). Gray: plasma membranes; Orange: synaptic vesicles; Cyan: presynaptic protein density; Magenta: PSD protein density; Green: actin filaments. (*C*) Quantification of 3D synapse ultrastructure, including cleft dimensions, AZ membrane area, synaptic vesicle number, and synaptic vesicle diameter (averaged per synapse, see *SI Appendix*, Fig. S1*D* for per vesicle diameters). Lines show mean ± SD (N = 14 synapses). (*D*) Histograms of the distance distribution of vesicles to the AZ membrane, normalized by AZ membrane area (N = 14 synapses). The main panel shows bins of 10 nm from 0 to 500 nm. The *Upper Right* *Inset* shows bins of 5 nm from 0 to 50 nm.

Synapses showed characteristic ultrastructure indicative of glutamatergic chemical synapses (Fig. 2 *A* and *B*). The presynaptic compartment was densely crowded with synaptic vesicles and numerous small protein densities. The postsynaptic compartment was in comparison sparser, with abundant branched actin filaments present throughout. The PSD was apparent as a dense pleomorphic network decorating the intracellular surface of the postsynaptic membrane in proximity to the synaptic cleft. This network is consistent with the “thick” PSDs found in previous lower-resolution cryo-ET studies of PSD-95 containing excitatory synapses (49). On one occasion, a “thin” PSD, corresponding to gephyrin containing inhibitory synapses, was also identified and excluded from further analysis [(49); *SI Appendix*, Fig. S2*A*]. On the presynaptic side of the synaptic cleft, supramolecular densities were apparent between membrane-proximal vesicles, reminiscent of previously described presynaptic dense projections proposed to be the morphological correlate of the AZ complex (28, 30). Additionally, we observed large membrane proteins on some vesicles consistent with V-ATPases, and other notable features such as postsynaptic clusters of putative TRiC (TCP-1 ring complex) and presynaptic clathrin baskets (*SI Appendix*, Fig. S2 *B–D*).

We quantified synaptic ultrastructure using three-dimensional reconstructed tomogram segmentation at a binned voxel size of 1.36 nm to assess how well our samples matched existing data on cultured neurons grown on glass coverslips. Our results are consistent with previous reports using tomography or serial section reconstructions of high-pressure frozen samples (33, 53, 54). The synaptic cleft was on average 24.4 nm in width (Fig. 2*C*) bordered by an AZ membrane area of 0.1 μm^2^. Synaptic vesicles had an average diameter of 48.7 nm and there was an average of 94 vesicles in the presynaptic terminal (Fig. 2*C* and *SI Appendix*, Fig. S1*D*). The number of synaptic vesicles is likely underestimated since the entire presynaptic terminal was not captured in the 3D field of view of every tomogram. We analyzed the distribution of distances between synaptic vesicles and the AZ membrane, normalized by membrane area and calculated as the number of synaptic vesicles per 0.1 μm^2^—the average AZ membrane area. Histograms with a bin-width of 10 nm showed a prominent peak in the 0-10 nm bin with a dip in the distribution in subsequent bins ranging from 10 to 50 nm (Fig. 2*D*). Using a smaller bin-width of 5 nm, the membrane-proximal bin (distance <10 nm) was split between synaptic vesicles 0 to 5 nm and 5 to 10 nm from the AZ membrane (Fig. 2 *D*, *Inset*). Previous serial section EM reconstructions of hippocampal neurons either cultured on glass coverslips or from tissue sections indicated a total AZ area of ~0.15 μm^2^ and ~12 docked synaptic vesicles per synapse (53, 54). Thus, our synapse tomograms from lamellae capture on average ~66% of the area of the synapse, assuming the membrane-proximal <10 nm vesicles (~8 vesicles/synapse; Fig. 2*D*) correspond to “docked” or “primed” vesicles.

### AZ and PSD Scaffolding Complexes Form Nanoclusters Detected by Cryo-ET.

Superresolution microscopy of fluorescently labeled proteins revealed nanoclusters of PSD and AZ proteins aligned trans-synaptically into nanocolumns (9, 10, 35, 36). However, using fluorescence microscopy alone is limited since only labeled proteins are localized, while membranes and the positions of membrane-proximal vesicles are not observable. Cryo-ET can overcome this limitation of fluorescence microscopy since it offers cellular context and molecular resolution. However, labeling and identifying specific proteins remains difficult, especially in crowded cellular environments. Thus, while we could not establish the precise localization of specific proteins, we reasoned that subsynaptic nanoclusters within the PSD and AZ could be apparent in our tomograms since these clusters line the synaptic plasma membrane, are tens of nanometers in diameter, and contain tens of copies of their respective constituent proteins with an approximate molecular mass in the megadalton range (9, 36).

We first adopted a masked autocorrelation approach to assess spatial clustering in specific synapse areas and to quantify the nanocluster dimensions. A volume band starting from the postsynaptic membrane and extending 100 nm into the postsynaptic compartment was used to encompass the PSD region (*SI Appendix*, Fig. S3*A*). A similar volume band extending 100 nm into the presynaptic terminal, excluding membrane-proximal synaptic vesicles, defined the AZ region. We used these volume bands as masks and quantified the normalized spatial autocorrelation within each mask based on voxel intensity values of Wiener-filtered tomograms. In the PSD region, autocorrelation values were above the expected random value [random normalized G(r) = 1] out to ~90 nm (*SI Appendix*, Fig. S3*B*). The AZ region showed similar autocorrelations to ~70 nm. These values are consistent with estimates of synaptic nanocluster dimensions ~80 nm (10, 35). To test that this type of analysis can measure the dimensions of subsynaptic features, we also performed autocorrelation analysis using the PSD region mask, but shifted into the synaptic vesicle cloud. The synaptic vesicle region showed peaks at ~40 and ~60 nm, consistent with the diameter of, and approximate spacing between, synaptic vesicles (Fig. 2*C* and *SI Appendix*, Figs. S3*B* and S4*E*).

**Fig. 3.**
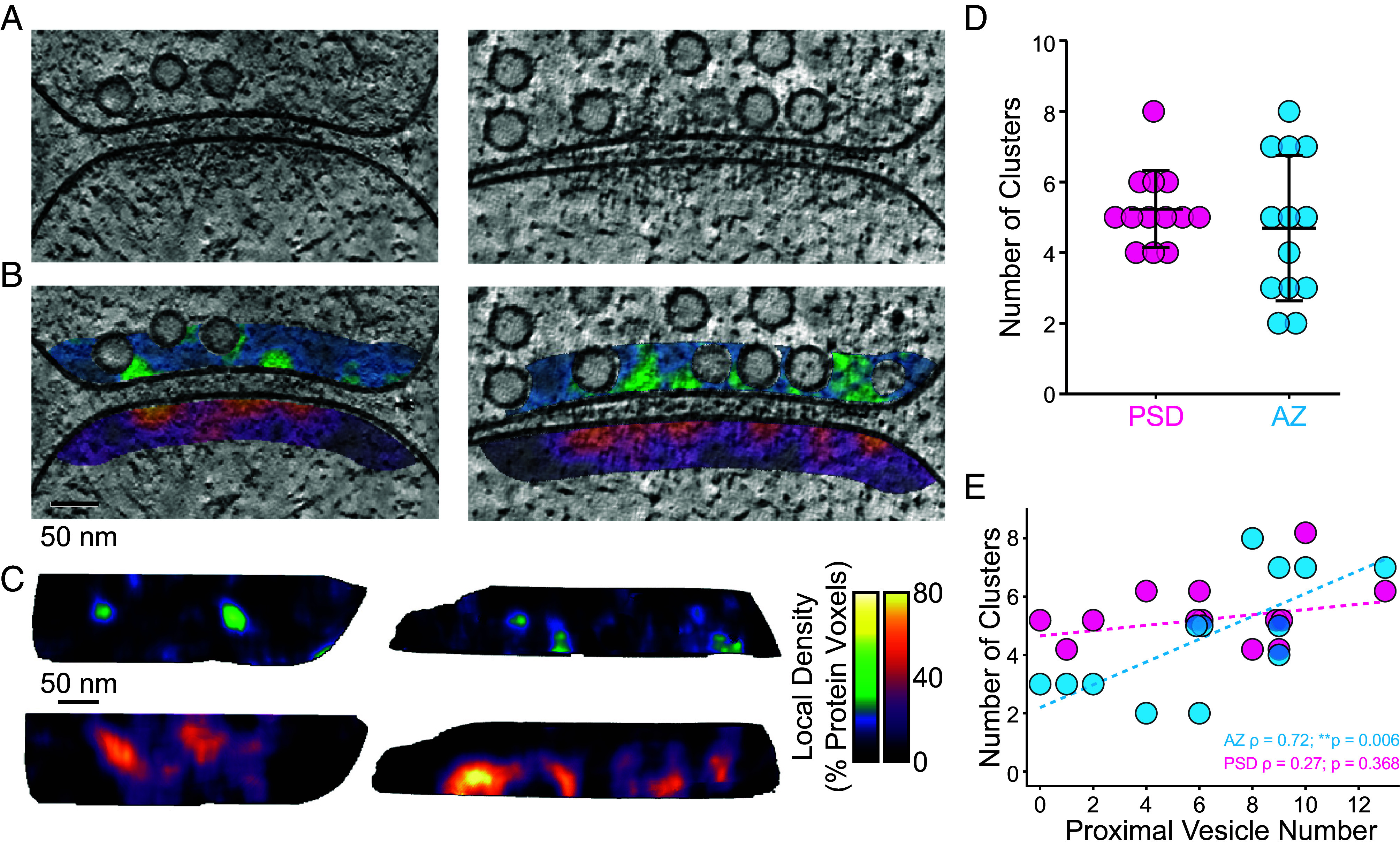
Nanoscale clustering of AZ and PSD scaffolding complexes. (*A*) Example XY slices of Wiener-deconvolved (63) tomograms used for segmentation-based local density analysis. (*B*) The same slices are shown in (*C*) with overlaid local density maps at 30% opacity. (*C*) Maximum intensity projections of local density maps corresponding to the example synapses shown in (*A* and *B*). Projections show the AZ on *Top* and the PSD on the *Bottom*, projected from the presynaptic perspective. (*D*) Quantification of the number of PSD and AZ protein nanoclusters within each synapse (N = 13 synapses). (*E*) Correlation between the number of membrane-proximal (<10 nm) vesicles and the number of PSD (magenta; Spearman rank correlation = 0.27, *P*-value 0.368) and AZ (cyan; Spearman rank correlation = 0.72, **P-value 0.006) protein nanoclusters (N = 13 synapses). Linear fits to each point set are shown in dashed lines. Spearman rank coefficients (ρ) and associated p-values are listed at the *Bottom Right* corner. Significance was determined by permutation testing.

To visualize scaffold nanoclusters directly, we applied a segmentation-based local density analysis. Voxels with intensity values 1.5 SDs above the mean of each region were segmented as protein voxels, and the local density was measured in a ~30 nm diameter window within each region. This analysis revealed nonuniform protein density peaks within the PSD and AZ regions, which visually corresponded to scaffolding complexes (Fig. 3 *A–C*). Maximum intensity projections onto the plane of the membrane showed a coarse correspondence between peaks in local density in the AZ and PSD (Fig. 3*C*). Peaks in local density maps were used as seed points for gradient-based segmentation of individual nanoclusters, thereby partitioning each local density map into discrete nanocluster volumes (*SI Appendix*, Fig. S3*C*). Nanoclusters identified in this manner likely represent large supramolecular clusters of proteins. AZ nanoclusters were on average 23,739 nm^3^ which could accommodate tens of copies of large proteins such as the Munc13. We quantified the number of nanoclusters per synapse and found an average of 5.23 PSD nanoclusters and 4.69 AZ nanoclusters, with higher variability in the number of AZ nanoclusters (PSD: SD = 1.05; AZ: SD = 1.98) (Fig. 3*D*). We then plotted the number of membrane-proximal vesicles versus either PSD or AZ cluster number for each synapse. There was a significant correlation between the number of membrane-proximal synaptic vesicles and the number of AZ nanoclusters per synapse (Spearman rank correlation = 0.72, **P-value 0.006) but not the number of PSD nanoclusters (Spearman rank correlation = 0.27, *P*-value 0.368) (Fig. 3*E*).

### Trans-Synaptic Alignment of Scaffolding Nanoclusters Does Not Extend to Synaptic Vesicles.

We next sought to quantitatively assess the alignment between AZ and PSD nanoclusters and membrane-proximal synaptic vesicles to test for the presence of nanocolumns and alignment with potential synaptic vesicle fusion sites. We developed an approach to measure the lateral offsets between clusters using the center of mass of each nanocluster volume projected onto the closest point on the postsynaptic membrane (*SI Appendix*, Fig. S3 *C–F*). We also projected the center of mass of each membrane proximal synaptic vesicle. For each nanocluster of a given type (i.e., PSD or AZ) the nearest neighbor distance between that cluster and a cluster of the opposite type was quantified. As a control, we randomly placed the same number of nanoclusters in the same area, repeating each simulated placement 1,000 times for each synapse.

**Fig. 4.**
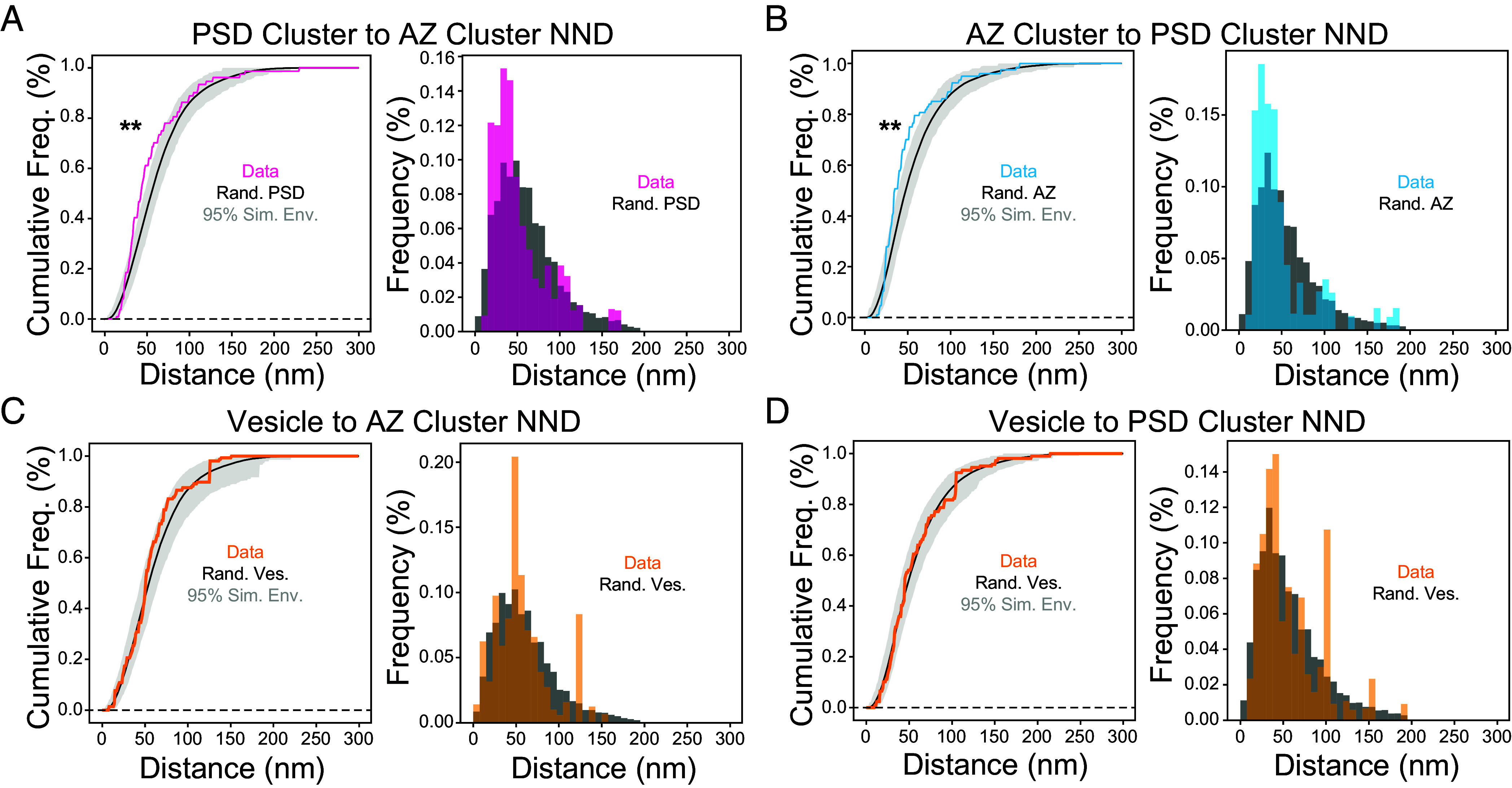
Quantification of trans-synaptic alignment of scaffold nanoclusters and membrane proximal synaptic vesicles. (*A*) Cumulative frequency (*Left*) and frequency (*Right*) histograms of the lateral distance between PSD clusters and their nearest neighbor AZ cluster (NND). The distribution of the observed distance data is shown in magenta compared to simulation means in black. The 95% simulation envelope of 1,000 simulated random PSD cluster positions is shown in gray (N = 68 PSD clusters, 13 synapses). (*B*) Cumulative frequency (*Left*) and frequency (*Right*) histograms of the lateral distance between AZ clusters and their nearest neighbor PSD cluster. Observed distance data are shown in cyan, and simulations in black/gray as in (*A*) (N = 61 AZ clusters, 13 synapses). (*C*) Cumulative frequency (*Left*) and frequency (*Right*) histograms of the lateral distance between synaptic vesicle centers and their nearest neighbor AZ cluster. Observed distance data are shown in orange, and simulations in black/gray (N = 85 vesicles, 12 synapses). (*D*) Cumulative frequency (*Left*) and frequency (*Right*) histograms of the lateral distance between vesicle centers and their nearest neighbor PSD cluster. Observed distance data are shown in orange, and simulations in black/gray as in (*A*) (N = 85 vesicles, 12 synapses). *P < 0.05; **P < 0.01 determined by MAD tests.

Comparing the distribution of nearest-neighbor distances (NND) between PSD clusters and AZ clusters revealed significantly shorter distances in the observed data than a randomized placement (observed median = 43 nm, simulated median = 55 nm) (Fig. 4*A*). Similarly, the distance between AZ and PSD clusters was shorter than that of randomized controls (observed median = 36 nm, simulated median = 47 nm) (Fig. 4*B*). These results are consistent with previous reports of trans-synaptic nanocolumns of AZ and PSD protein nanoclusters (10). Next, we measured the alignment between membrane proximal synaptic vesicles and AZ/PSD nanoclusters. Surprisingly, we found no difference from randomized synaptic vesicle placement in the distance between vesicles to AZ nanoclusters (observed median = 51 nm, simulated median = 54 nm) or to PSD nanoclusters (observed median = 45 nm, simulated median = 49 nm) (Fig. 4 *C* and *D*). Nearest neighbor distances between clusters of the same type and between synaptic vesicles were no different from random (*SI Appendix*, Fig. S4 *C–E*). We also examined a subset of AZ nanoclusters which formed trans-synaptic nanocolumns. Nanocolumns were defined as AZ and PSD nanoclusters that were each other’s nearest neighbor and were within 100 nm of one another. There was no preferential alignment of synaptic vesicles to these nanocolumns (*SI Appendix*, Fig. S5*F*). Together, we observe preferential alignment between nanoclusters of AZ and PSD scaffolding proteins and random placement of membrane-proximal synaptic vesicles with respect to AZ and PSD nanoclusters.

### Pleomorphic Protein Densities Tether Membrane Proximal Synaptic Vesicles.

**Fig. 5.**
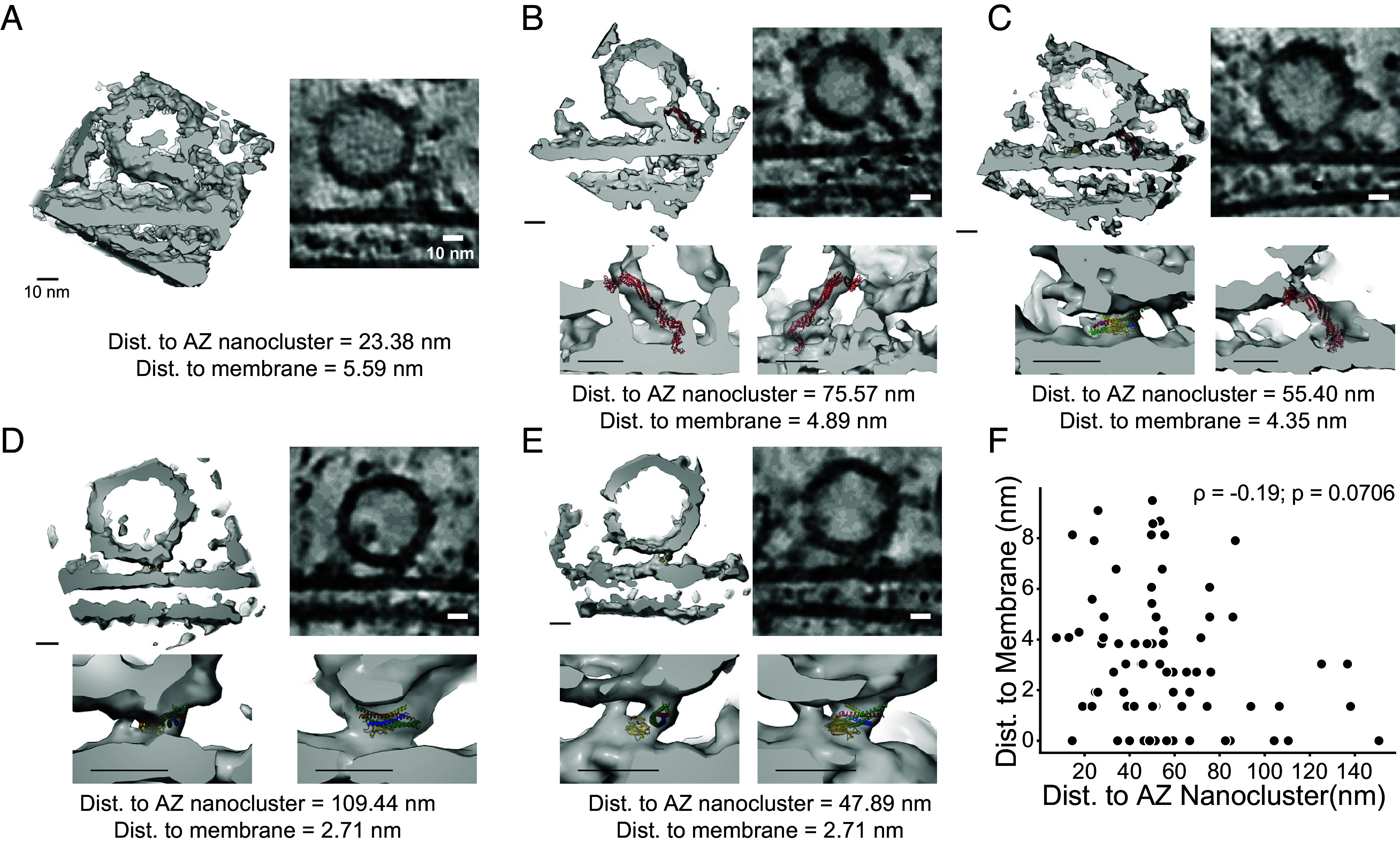
Protein densities surrounding membrane proximal synaptic vesicles. (*A*) Example of a synaptic vesicle in apparent contact with an AZ supramolecular nanocluster. The volume is shown as a 3D surface rendering (*Left*) and a single XY tomogram slice 1.36 nm thick (*Right*). The distance between the vesicle and plasma membrane and the lateral distance between the vesicle and AZ nanocluster center-of-mass are listed below. (*B*) Example of a comparably simple interface with an apparent tether. The atomic model of the C_1_-C_2_B-MUN-C_2_C fragment in the upright conformation is shown in red [(64); PDB 7T7X]. (*C*) Example of an interface with both tether and intermembrane globular densities. Atomic models: red = C_1_-C_2_B-MUN-C_2_C fragment in the lateral conformation [(64); PDB 7T7V]; SNARE/Syt1-C_2_B primary interface [(65); PDB 5W5C], red = syntaxin, green = SNAP25, blue = synaptobrevin, yellow = complexin, gold = Syt1-C_2_B. (*D* and *E*) Examples of interfaces with intermembrane globular density but no apparent tether. Atomic models: SNARE/C_2_B primary interface [(65); PDB 5W5C]. (*F*) Relationship between vesicle-to-plasma membrane distance and vesicle-to-AZ nanocluster distance for all vesicles in the dataset (N = 85 vesicles, 12 synapses; Spearman rank correlation = −0.19, *P*-value 0.0706). All scale bars are 10 nm.

Nanoclusters of AZ proteins, including Munc13 and RIM, are correlated to functionally defined release site number and position (10, 36). These correlations are based on functional experiments where action potentials evoke single vesicle fusion events. On the other hand, our cryo-ET experiments analyzed all membrane-proximal vesicles within 10 nm of the plasma membrane. We therefore asked whether additional morphological features could be used to identify subsets of synaptic vesicles in different states. To examine each synaptic vesicle more closely, we extracted denoised subtomograms of membrane-proximal vesicles. We processed them by Wiener filtering to alleviate contrast transfer function artifacts and reduce high-frequency noise (62, 63) (Fig. 5). Previous cryo-ET studies of synaptosomes revealed tethers linking membrane-proximal synaptic vesicles to the AZ membrane (44–47). In agreement with this previous work, all membrane-proximal synaptic vesicles were connected to the membrane by proteinaceous densities in our tomograms. However, these densities were highly variable, and we could not detect stereotyped architecture. In some cases, synaptic vesicles appeared to make direct contact with AZ nanoclusters (Fig. 5*A*). Other synaptic vesicles were more distal from nanoclusters and were tethered to the plasma membrane by elongated densities at the periphery of the vesicle. These elongated tethers were roughly consistent in size and shape with isolated copies of the priming protein Munc13 and could accommodate Munc13 in different conformations of the C1-C2B hinge region (64) (Fig. 5 *B* and *C*). Synaptic vesicles closer to the membrane had globular densities near the point of closest membrane approach, either with or without additional elongated densities (Fig. 5 *C–E*). These globular densities between the synaptic vesicle and the plasma membrane are consistent in volume and shape with models of the complex of the C2B domain of synaptotagmin-1 and the SNARE complex interacting via the primary interface and complexin (65); PDB ID 5W5C). This supports the notion that the final stage of synaptic vesicle maturation involves tight docking to the plasma membrane and the formation of a stably primed trans-SNARE complex (33, 65, 66). However, there was no correlation between synaptic vesicle distance to the plasma membrane and proximity to AZ nanoclusters (Fig. 5*F*). Thus, AZ nanoclusters have no clear spatial relationship with membrane-proximal synaptic vesicles in different putative priming states.

### Random Alignment of Vesicle Fusion to PSD Nanoclusters Increases Synaptic Response Variability.

**Fig. 6.**
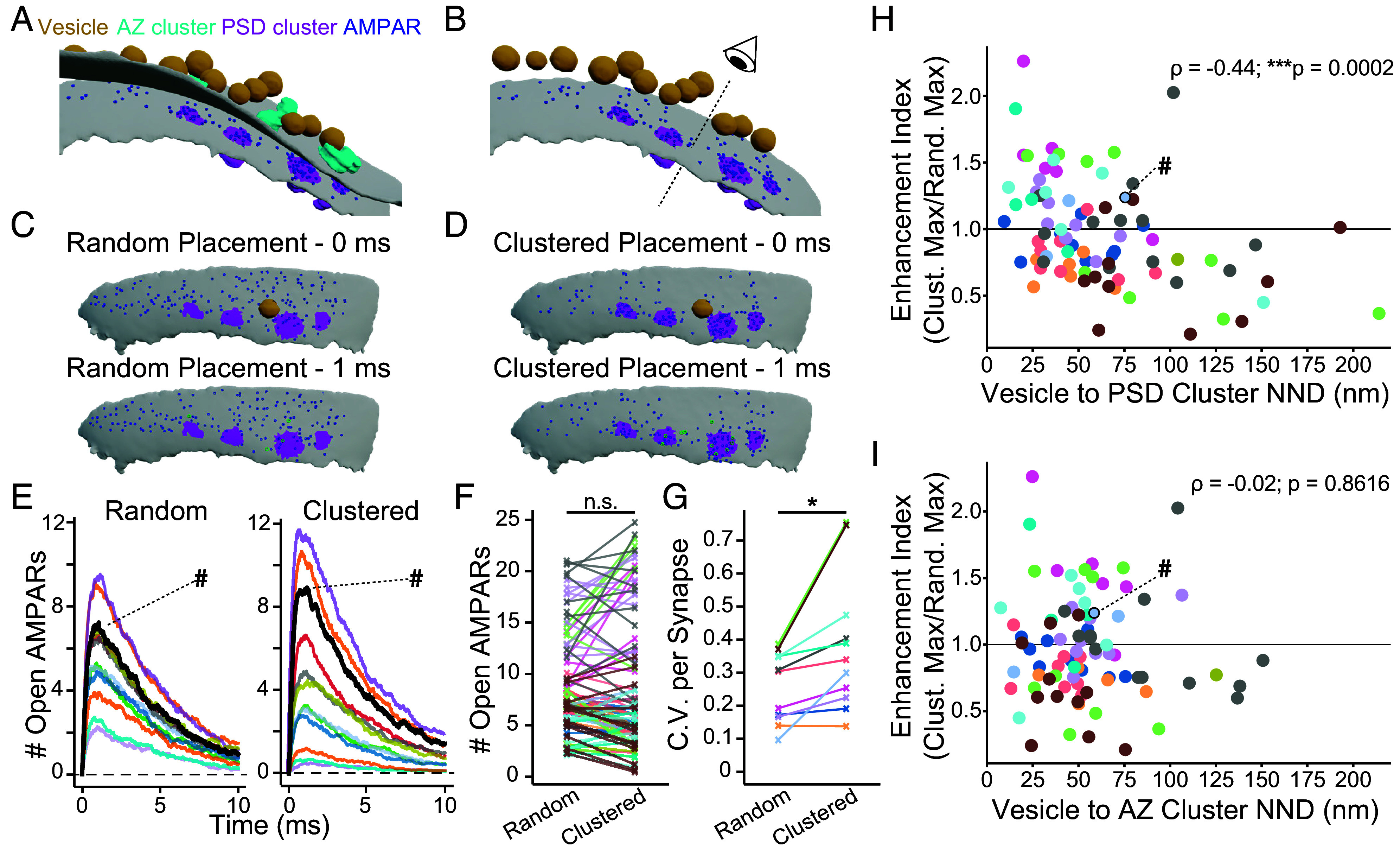
Tomogram-guided simulations of single vesicle fusion events. (*A*) 3D rendering of a simulation environment derived from a tomogram segmentation. Plasma membranes are shown in gray, synaptic vesicles in orange, AZ nanoclusters in cyan, PSD nanoclusters in magenta, and AMPA receptors in dark blue. (*B*) The same model as in A, but with the presynaptic plasma membrane and AZ nanoclusters removed. The eye indicates the perspective—looking down from the vesicle cloud to the postsynaptic membrane—of the images in (*C* and *D*). (*C*) Snapshots of simulation time points at 0 ms (*Top*) and 1 ms (*Bottom*) showing receptors seeded in the random configuration throughout the PSD region of the postsynaptic membrane. For clarity, the synaptic vesicles are omitted except for the fusing vesicle (shown in the *Top* panel in *C*). AMPA receptors that have opened in response to glutamate release are shown in green at the 1 ms time-point. (*D*) Snapshots of simulation time points at 0 ms (*Top*) and 1 ms (*Bottom*) showing AMPA receptors seeded in the clustered configuration. The same synaptic vesicle fusion event occurs as in (*C*), and open AMPA receptors are shown in green at the 1 ms time-point. (*E*) The number of open AMPA receptors versus time in the random (*Left*) and clustered (*Right*) configurations are plotted for every vesicle of the synapse shown in (*A*–*D*). The bold black line marked with ‘#’ indicates the vesicle shown in panels (*C* and *D*). Traces are color-coded by vesicle. (*F*) The maximum number of open AMPA receptors in response to single vesicle fusion events with receptors in random versus clustered configurations. The plot shows values for all membrane-proximal vesicles of all synapses in the dataset with lines and markers color-coded by synapse. (*G*) Coefficient of variation (C.V.) values for each synapse, color-coded as in (*F*). *P < 0.05 as determined by the paired *t* test. (*H*) Relationship between the vesicle-to-PSD nanocluster distance and the enhancement index for each vesicle in the dataset. The enhancement index was defined as the maximum number of open AMPARs in the clustered configuration normalized to the random configuration, with values greater than one indicating increased postsynaptic responses due to AMPAR clustering and values less than one indicating decreased responses. Points represent each vesicle, color-coded by synapse as in (*F* and *G*). The point marked with ‘#’ indicates the vesicle shown in panels (*C* and *D*). The Spearman rank coefficient (ρ) and associated p-value are listed at the *Top Right* corner (N = 85 vesicles, 12 synapses; Spearman rank correlation = −0.44, ***P < 0.001, as determined by permutation testing). (*I*) Relationship between the vesicle-to-AZ nanocluster distance and the enhancement index for each vesicle in the dataset. The Spearman rank coefficient (ρ) and associated *P*-value, as determined by permutation tests, are listed at the *Top Right* corner (N = 85 vesicles, 12 synapses; Spearman rank correlation = −0.02, *P*-value 0.8616).

The observed lack of preferential alignment between membrane-proximal synaptic vesicles and AZ or PSD nanoclusters was surprising, considering the potential importance of such alignment for AMPAR activation during synaptic transmission. To understand possible functional consequences of our findings, we performed Monte Carlo simulations of synaptic vesicle fusion events and subsequent AMPA receptor activation (opening) within a simulated synaptic cleft. We used our tomogram segmentations to generate biorealistic models incorporating the measured positions of synaptic vesicles, pre- and postsynaptic membranes, as well as AZ and PSD nanoclusters (Fig. 6 *A* and *B*). AMPA receptors were placed into the PSD membrane in two alternative topographies—random or clustered—at an overall density of 1,500 receptors/μm^2^. In the random configuration, receptors were distributed throughout the PSD membrane with no regional preference (Fig. 6*C*). In the clustered configuration, 75% of receptors were seeded into membrane patches aligned with PSD nanoclusters as observed in a particular tomogram, and the remaining 25% were placed randomly throughout the remaining PSD membrane [(67); Fig. 6*D*]. Fusion events were simulated for each membrane proximal synaptic vesicle in a synapse to compare the number of AMPA receptors activated in the clustered versus random configuration (Fig. 6*E*).

Comparing all synaptic vesicles, there was no difference in the mean number of open receptors between conditions (Fig. 6*F*). The simulated data from individual synaptic vesicles revealed that while some synaptic vesicle responses were strongly potentiated by clustered AMPA receptor topographies, other vesicle responses were reduced. When we examined the intervesicle variability in response amplitude, we found that the coefficient of variation (C.V., the ratio of the SD to the mean) within a synapse is increased by placing AMPARs in a clustered topography (Fig. 6*G*). We measured the impact of clustering by calculating an enhancement index for each synaptic vesicle as the ratio of the peak response amplitude in the clustered versus the random configuration. A value above one indicates a potentiation of the response due to AMPAR clustering, whereas a value below one indicates a reduction. As expected, there was a negative correlation between the enhancement index and vesicle distance to the nearest PSD nanocluster (Fig. 6*H*). In contrast, there was no such relationship between the enhancement index and distance to the nearest AZ nanocluster (Fig. 6*I*). There was a significant correlation between the enhancement index and vesicle distance to a nanocolumn (*SI Appendix*, Fig. S5*G*). However, simulating fusion events for only nanocolumn-associated synaptic vesicles - those with nearest-neighbor AZ nanoclusters that were part of a nanocolumn - gave similar results, with no increase in simulated response amplitude in the clustered versus random AMPAR configuration and an increase in response amplitude C.V. in the clustered configuration (*SI Appendix*, Fig. S5*H*).

Synaptic vesicles can be released both synchronously and asynchronously in response to action potentials (68). Release sites for synchronous and asynchronous release are proposed to be spatially segregated, with asynchronous fusion preferentially occurring at the center of the synapse and synchronous fusion occurring peripherally (69, 70). Additionally miniature release in the absence of action potentials is thought to occur at distinct release sites (71). We therefore examined whether the position of synaptic vesicles within the synapse correlated with their alignment to AZ or PSD nanoclusters. Both AZ and PSD nanoclusters were randomly localized relative to the synapse center (*SI Appendix*, Fig. S5 *A* and *C*). There was no correlation between the distance of a synaptic vesicle to the synapse center and its distance to the nearest AZ nanocluster (*SI Appendix*, Fig. S5*B*). There was a slight but significant positive correlation (Spearman rank correlation = 0.26) between vesicle distance to the synapse center and alignment to PSD nanoclusters (*SI Appendix*, Fig. S5*D*). However, our simulations suggest that there is no correlation between release amplitude enhancement index and synaptic vesicle distance to the synapse center (*SI Appendix*, Fig. S5*E*). Our tomogram-guided simulations demonstrate a surprising consequence of protein clustering at the synapse when contextualized within the cellular ultrastructure. If AMPARs adopt a clustered topography that is random relative to the placement of synaptic vesicles, even within a single synapse, some synaptic vesicles are predicted to have greater postsynaptic weight than others (Fig. 4 *C* and *D*; Fig. 6 *E*–*H*).

## Discussion

Our work demonstrates the utility of FIB milling of cultured neurons, building on work in synaptosomes and nonmilled cultures (44–49). FIB milling dramatically improved image contrast of our samples by reducing sample thickness to ~150 to 200 nm (Fig. 2 and *SI Appendix*, Fig. S1). This allowed us to directly visualize fine ultrastructural features of the synapse in detail. We observed supramolecular clusters of AZ and PSD scaffolds on the scale of tens of nanometers and trans-synaptic alignment of said nanoclusters (Figs. 3 and 4), consistent with previous studies using superresolution fluorescence microscopy (9, 10, 35, 36). Beyond these studies, we surprisingly found that membrane-proximal synaptic vesicles showed no preferential alignment with AZ or PSD nanoclusters, nor nanocolumns (Fig. 4 and *SI Appendix*, Fig. S5*F*). Instead, synaptic vesicles within 10 nm were linked to the membrane by elongated tether proteins or intermembrane globular protein densities, going beyond findings in lower-resolution tomograms of synaptosomes (44–47). These proteinaceous densities were not stereotyped and had no apparent relationship with AZ nanoclusters (Fig. 5). Tomogram-guided simulations of synaptic transmission suggested that the observed nanoscale topography drives an increase in synaptic response variability within a synapse, with postsynaptic response amplitude to single vesicle fusion events weighted by vesicle proximity to PSD nanoclusters (Fig. 6).

Their relatively small size (~0.5 to 1 μm diameter) and distal positioning from the soma make synapses “thin enough” for cryo-ET. Although cryo-ET imaging of nonmilled samples increases data collection throughput, the thickness of these samples is substantially larger than the mean free path of inelastic scattering, reducing tomogram quality and contrast. Synaptosome preparations, which shear synapses from the cell body, are necessarily harsh. Likewise, imaging nonmilled synapses from cultured neurons is limited to thin regions isolated over foil holes. These synapses are likely compressed during blotting and potentially experience substantial shear force (72). Targeting synapses within neurite bundles for subsequent FIB milling, therefore, offers notable advantages for the study of ultrastructure. Our tomograms indicate that lamella samples capture substantial portions of the total synaptic volume and show ultrastructure similar to synapses from cultures grown on glass coverslips. The tradeoff between cellular content lost during FIB milling and improved image quality supports FIB-milled samples as the sample of choice for high-resolution analysis of synapses by cryo-ET.

Using cryo-ET to visualize nanoscale clusters of PSD and AZ scaffolding complexes (Fig. 3) allowed us to analyze their topographical relationships to synaptic vesicles, providing context to the model of trans-synaptic alignment of neurotransmitter release and detection at glutamatergic synapses (9, 10, 35, 36). Fluorescence microscopy has shown that AZ nanoclusters of Munc13 contain tens of protein copies and are numerically correlated with functionally defined release site number, with an average of 5.7 clusters per synapse (36). Synaptic vesicle fusion events occur in clusters, suggesting a higher cluster number of 8.7 per synapse (38). Indeed, our tomograms support a positive correlation between the number of AZ nanoclusters and the number of membrane-proximal synaptic vesicles per synapse (Fig. 3*E*). Surprisingly, AZ nanoclusters showed no preference for alignment with synaptic vesicles, and they would likely occlude direct contact between the vesicle and plasma membranes (Figs. 4 and 5). One possibility is that AZ nanoclusters act at an upstream step to capture synaptic vesicles and allow the formation of initial membrane tethering interactions by Munc13 (73). These vesicles could then laterally disperse from AZ nanoclusters in contact with Munc13 molecules and proceed along the molecular priming process. This model would preserve the numerical correlation between, for example, clusters of Munc13 and the number of release sites but argue against the notion that those clusters precisely marked the position of release sites. While these nuances may only amount to shifts of tens of nanometers, such distances can potentially impact downstream signal transduction (8, 74). Alternatively, release sites that preferentially participate in a specific release modality (i.e., synchronous, asynchronous, or miniature release) may be preferentially aligned to AZ nanoclusters. Our data do not allow us to infer vesicular release probability. Still, even assuming synaptic vesicle distance to AZ nanoclusters correlates to high release probability, our simulations suggest that this does not reliably translate into an increased AMPAR response amplitude (Fig. 6*I*). AZ proteins form multiple interactions observed in vitro, colocalize with one another at the synapse, are involved in localizing one another, and form dense projections similar in appearance to the AZ nanoclusters we visualize by cryo-ET (25, 29, 30, 32, 34). However, it remains possible that distinct AZ subcomplexes exist and that parsing these could reveal specific alignments obscured in our current analysis. Currently, we can only select for nanoclusters that are part of trans-synaptically aligned nanocolumns. In any case, we did not observe a preferential alignment of nanocolumns with synaptic vesicles. Moreover, we did not observe an enhancement of the simulated response by clustered AMPARs when averaged across all nanocolumn-associated synaptic vesicles (*SI Appendix*, Fig. S5*H*).

Subtomogram volumes extracted from our samples show improved image contrast compared to previous nonmilled samples, resolving membrane leaflets and complex protein densities surrounding membrane-proximal synaptic vesicles (*SI Appendix*, Fig. S1 *A–C*). We observed elongated tether densities consistent with upright and lateral conformations of the C_1_-C_2_B-MUN-C_2_C fragment of Munc13 (64) (Fig. 5 *B* and *C*). Membrane-proximal synaptic vesicles were found without putative Munc13 densities (*SI Appendix*, Fig. S5 *D* and *E*), associated with one or two (Fig. 5 *B* and *C*), or associated with large AZ nanoclusters of sufficient size to accommodate tens of copies of proteins the size of Munc13 (Fig. 5*A*). This suggests that large AZ nanoclusters do not correlate precisely with vesicle position within the synapse. Additionally, we observed globular densities near the site of closest approach between the synaptic vesicle and the plasma membrane (Fig. 5 *C*–*E*). These globular densities are consistent with complexes of SNAREs, synaptotagmin, and complexin. These complexes are sufficiently stable to be visible in our cryo-ET tomograms, supporting the existence of prefusion complexes that juxtapose membranes in an inhibited conformation at resting Ca^2+^ concentration (65, 66, 75). However, we did not observe symmetric protein architecture surrounding the synaptic vesicle. As a result, definitively confirming the molecular identity of these densities via in situ structure determination will likely require labeling or genetic approaches (e.g., knock-out) in addition to high-throughput data collection.

The physiological impact of clustered topographies of scaffolding proteins and receptors crucially depends on the ultrastructural context. Signaling through AMPA receptors depends on relative positioning between synaptic vesicle fusion sites and receptor clusters (4, 8, 9, 11, 76). Our tomograms revealed that membrane-proximal synaptic vesicles are not preferentially aligned with nanocolumns of AZ and PSD scaffolds (Fig. 4). Our tomogram-guided Monte Carlo simulations suggest that the functional consequence is increased postsynaptic response variability within single synapses. While some vesicles were well aligned to AMPARs within nanoclusters and thereby had potentiated postsynaptic responses, others were effectively sequestered and thereby had their response amplitude inhibited (Fig. 6*H*). This is consistent with previous modeling work on sources of variability in quantal response amplitude, which postulated the existence of silent subregions of the synapse (8). Previous work analyzing AMPAR response amplitude C.V. revealed values ranging from 0.28 in high calcium solutions to 0.36 and 0.55 when release was stimulated with hypertonic sucrose (3, 77, 78). Our simulation average C.V. was 0.38 for clustered AMPARs and 0.26 for random AMPAR placement. The C.V. values in the clustered configuration closely match published data and represent the variability expected when all membrane-proximal synaptic vesicles are allowed to fuse, which closely approximates sucrose stimulation (3, 77). While synaptic vesicles are not preferentially aligned to AZ nanoclusters, fusion events are known to be predicted by the local density of the AZ scaffold RIM, which forms nanocolumns with PSD-95 (10). This suggests a correlation between vesicular release probability and alignment with PSD nanoclusters, wherein well-aligned vesicles are most likely to fuse during an action potential. We speculate that tuning alignment may serve as an axis for synaptic plasticity or that synaptic vesicles that are “misaligned” may target alternative receptor types (10, 69).

## Methods

### Neuronal Culture Preparation.

All animal experiments were performed in accordance with Stanford APLAC institutional guidelines. Primary cultures of mouse hippocampal neurons were prepared following published protocols (53, 79). Briefly, gold 200 mesh Quantifoil R2/2 or R1/4 grids were glow discharged (15 mA, 45 s) and then placed in 35 mm glass-bottom dishes in a biosafety cabinet under UV illumination for 30 min. Grids were then coated in Matrigel for 1 h at 37 °C before use. Postnatal day 0 (P0) pups of wild-type C57BL6/J mice were anesthetized on ice before decapitation and bilateral dissection of the hippocampus. Hippocampi were treated with a papain solution (10 mL Hank’s Balanced Salt Solution (HBSS), 10 µL 0.5 M EDTA pH 8.0, 10 µL 1 M CaCl2, 100 µL papain, 100 µL DNAse I) for 15 min at 37 °C, prior to gentle trituration to generate a single cell suspension. Cells were resuspended in 1 mL per pup of plating medium [Minimum Essential Medium (MEM) with 0.5% glucose, 0.02% NaHCO3, 0.1 mg/mL transferrin, 10% Fetal Select bovine serum, 2 mM L-glutamine, and 25 mg/mL insulin]. Excess Matrigel solution was removed from the grids, and cell suspension was bubbled on top of each grid for 45 min, followed by flooding of the entire dish with growth medium. After 1 d in vitro (DIV1), medium was exchanged to growth medium composed of MEM with 0.5% glucose, 0.02% NaHCO3, 0.1 mg/mL transferrin, 5% fetal bovine serum, 2% B-27 supplement, and 0.5 mM L-glutamine. At DIV3-5, half the medium was exchanged to a growth medium supplemented with 4 μM Cytosine b-D-arabinofuranoside (AraC) to inhibit glial cell division. Cultures were maintained until DIV14-16 before vitrification to allow synapses to form and reach functional maturity (79). Grids were frozen using a Leica EMGP plunge freezer set to 25 °C and 95% humidity. Grids were blotted from the back side—the side opposite the adherent neurons—for 5 s, then immediately plunged into liquid ethane. Vitrified grids were clipped into cryo-FIB autogrids before FIB milling and TEM tilt-series data collection.

### Cryo-FIB Milling.

Vitrified cultured neurons were loaded into an Aquilos 2 cryo-FIB-SEM system cooled to −190 °C in a 45-degree pretilt shuttle. Overview images of each grid were acquired at a stage tilt of 16° before sputter and GIS coating. This facilitated the identification of neurite bundles that often have relatively flat profiles, making them difficult to identify from perspectives perpendicular to the grid plane. Samples were then sputter coated with platinum for 15 s at 30 mA and 0.10 mBarr (rough coating), followed by GIS coating for 15 s at an orientation perpendicular to the grid plane. Regions of interest were identified from overview scans where bundles of neurites appeared to connect cell soma in neighboring grid squares. Higher magnification SEM scans were used to identify characteristic features of neurite bundles, including rough texture with visible varicosities. Lamellae were manually milled at a 9-degree milling angle using rectangle patterns between 15 and 17 μm in X width. The milling sequence was the following (all at 30 keV): rough milling—0.3 nA, 3 μm pattern separation in Y; medium milling—0.1 nA, 1 μm pattern separation; fine milling—50 pA, 500 nm pattern separation; polishing—30 pA, 175 nm pattern separation. During polishing, endpoint monitoring was performed using intermittent SEM scans of the lamella at 3 keV. Once charging contrast was lost, or if the GIS layer was milled through, polishing was stopped (58).

### Tilt-Series Data Collection and Tomogram Reconstruction.

Milled grids were loaded onto Titan Krios TEM microscopes equipped with a Gatan energy filter and Gatan K3 direct electron detector. Tilt-series data were collected using SerialEM in low-dose mode at a physical pixel size of 1.735 Å/px. The energy filter slit-width was set to 20 eV. Tilt series were collected in three-degree increments to ±60 degrees, starting at a 9-degree tilt to offset the tilt of the lamellae. A dose-symmetric tilt scheme was used with a grouping of two (tilt sign inversions every other step) (80). The dose per tilt was 3.2 e/Å^2^, resulting in a total dose of 131.2 e/Å^2^ over 41 tilts. Images were collected as dose-fractionated movies with a per-frame dose of 0.21 e/Å^2^. Motion and gain correction was performed in Warp (63), along with initial CTF estimation. Even and odd motion-corrected half averages of each tilt image were saved for later use in denoising. Tilt-series stacks were reconstructed in Warp and used for CTF estimation and fit quality estimates (*SI Appendix*, Fig. S2*C*). Tilt-series stacks were aligned in IMOD using surface contamination features as fiducial markers (81). Aligned stacks were binned by eight to a pixel size of 1.36 nm, and tomograms were reconstructed in IMOD using weighted backprojection. The full dataset included 13 tomograms and 14 synapses (i.e. one tomogram captured two synapses) with one additional putative inhibitory synapse tomogram that was excluded from analysis.

### Ultrastructure Analysis.

For analyzing synapse ultrastructure, tomograms were first denoised with cryoCARE using identically reconstructed “half-tomograms” (62). Tomogram segmentations were performed using several different algorithms, as detailed below, and the results imported into Amira. First, membrane segmentation was performed in Amira using a combination of the membrane enhancement filter and manual tracing (82). Synaptic vesicles were automatically segmented in EMAN2 using a convolutional neural network trained on a subset of manually annotated vesicle cross-sections (83). Actin filaments were traced using the cylinder correlation and fiber tracing modules in Amira (84). To define the boundaries of the synaptic cleft, a distance field from the postsynaptic membrane was calculated for each tomogram. This assigns each voxel a value of its distance to the membrane. This distance field was multiplied by the binary segmentation of the presynaptic membrane to determine the distance of each presynaptic membrane voxel from the postsynaptic membrane. Presynaptic membrane voxels greater than 10 nm and less than 40 nm from the postsynaptic membrane were considered part of the AZ membrane. The mirror of this operation—calculating a distance field for the presynaptic membrane and assigning postsynaptic voxels distances—was used to determine the PSD membrane region. The cleft width was measured as the average distance between the AZ and PSD membrane regions. The PSD region was defined as the postsynaptic intracellular space less than 100 nm from the PSD membrane. Similarly, the AZ region was defined as the presynaptic intracellular space less than 100 nm from the AZ membrane, with the volume of any membrane proximal synaptic vesicles subtracted. Vesicle diameters were calculated using vesicle segmentations as the equivalent diameter of a sphere of the same volume as each segmented vesicle. Intervesicle distances were calculated as the nearest neighbor distance between the center of mass of each vesicle. For visualization in Fig. 2, equivalent diameter spheres were placed at the same center of mass location as each segmented vesicle. The vesicle distances to the AZ membrane were determined by multiplying the original binary vesicle segmentations by the AZ distance field and taking the minimum voxel distance value for each vesicle.

### Visualization of Proximal Vesicle Subtomograms and Docking of Munc13-1/Primed SNARE Complex Structures.

To extract membrane-proximal synaptic vesicles, the coordinates of the closest voxel to the membrane for each vesicle were taken as the center of the extraction volume for each vesicle less than 10 nm from the AZ membrane. Subtomograms (60 × 60 × 60 voxels) at 1.36 nm/voxel were extracted using IMOD (81) from Wiener-filtered tomograms and visualized in ChimeraX (63, 85). Three atomic models were used for docking into putative protein densities: the primed SNARE complex with synaptotagmin C2B in the primary interface and the complexin helix from the tripartite interface [(65); PDB 5W5C], the lateral conformation of the C_1_-C_2_B-MUN-C_2_C fragment of Munc13-1 [(64); PDB 7T7V], and the upright conformation of the C_1_-C_2_B-MUN-C_2_C fragment of Munc13-1 [(64); PDB 7T7X]. A cylindrical mask was used for each protein density to isolate the protein region from the surrounding membranes. Some densities could be missed due to the missing wedge effect, especially when they are located at positions and angles that would require viewing in the direction of the missing wedge. Atomic models were manually docked in accordance with the known orientation of each protein, syntaxin, and SNAP25 helices were oriented toward the plasma membrane and synaptobrevin toward the synaptic vesicle. Munc13-1 C_1_-C_2_B domains were likewise placed near the plasma membrane and C_2_C toward the vesicle membrane. After manual placement, the fit function of ChimeraX was used to place each model in the respective density. Two tomograms were excluded from this analysis; one due to the presence of prominent lamella surface contamination, which generated periodic streaking artifacts throughout the tomogram, and a second which had no membrane-proximal vesicles.

### Autocorrelation Analysis.

To perform masked autocorrelation analysis, tomograms were first processed by Wiener filtering (63), then normalized to 16-bit with inverted contrast, i.e., bright signal dark background. Mask regions were generated for each area of interest, namely the PSD and AZ regions, as defined above in the section on ultrastructure analysis. For the vesicle cloud region, the mask used to define the PSD region was shifted into the vesicle cloud. Autocorrelations were performed using fast Fourier transforms on masked regions and normalized to the autocorrelation of the mask multiplied by the average voxel value of the image within the mask. This normalization accounts for the shape of the mask region itself so that elevated autocorrelation values (above one) reflect spatial structure within the masked region. Normalized autocorrelation volumes were then averaged in spherical shells to plot the 1D autocorrelation versus shell radius. All analysis was implemented in Python.

### Local Density Analysis.

Segmentation-based local density analysis was performed for the PSD and AZ regions using tomograms deconvolved with a Wiener filter (63). For each region, a threshold of 1.5 SDs above the mean voxel intensity value was used to define and segment protein density as “PSD material” or ‘AZ material.’ Boundary-corrected local density analysis was performed in Amira using a window of 21 voxels (28.6 nm). The window value was set based on the spatial dimensions identified using autocorrelation analysis. This generated local density maps with each voxel assigned a value of the percentage of protein voxels within the window region. From these density maps, voxels 2.5 times the mean local density were used as seed points to segment scaffold clusters using the “propagating contour” function in Amira. This function computes a gradient image from the grayscale input values, in this case, local density, and propagates a segmentation starting from seed points with the propagation velocity dictated by the gradient image. For all tomograms the following settings were used: propagation time 10, edge sensitivity 0.5, image intensity weight 0.5. These settings were chosen based on manual inspection of segmentations of a subset of tomograms such that visually separable peaks in local density were not merged. The center of mass of each segmented cluster was then calculated and used as the cluster center in subsequent analysis. Visualization and maximum intensity projections of local density maps were performed by either FIJI or Napari (86). One tomogram used for ultrastructure analysis in Fig. 2 was excluded from local density analysis due to the presence of prominent lamella surface contamination, which generated periodic streaking artifacts throughout the tomogram.

### Cluster Nearest-Neighbor Distance Analysis.

To analyze lateral offsets between scaffolding clusters and vesicles, cluster center coordinates, and synaptic vesicle center coordinates were projected onto the closest point on the postsynaptic membrane. The Euclidean distance between points could then be calculated to primarily reflect lateral displacement along the plane of the synaptic cleft. For each cluster, the nearest-neighbor distance to a cluster of the opposite type (i.e. PSD to AZ, AZ to PSD) or a vesicle center was calculated using custom Python scripts. As a control, for each synapse, 1,000 simulated cluster centers were generated with the same number of clusters as the original data, and the minimum spacing between like clusters (i.e., PSD to PSD cluster distance) was set to match the observed data. Only one type of cluster was randomized at a time. For analyzing the distance of each synaptic vesicle to the synapse center, the synapse center was approximated as the center of mass of the PSD membrane region as defined above for ultrastructural analysis. This is only an estimate as the removal of material during FIB milling could remove the true center of mass of the synapse as a whole. Nanocolumns were defined at AZ and PSD nanoclusters that were each other’s nearest neighbor and were within 100 nm of one another.

### Monte Carlo Reaction–Diffusion Simulations.

Monte-Carlo simulations of synaptic vesicle fusion events and subsequent AMPA receptor activation were performed using MCell and Cellblender (87–89). Segmentations of tomograms, including pre- and postsynaptic plasma membranes, membrane-proximal synaptic vesicles, and AZ and PSD scaffold clusters were imported into Blender as triangle mesh surfaces. Vesicle fusion events were simulated as the point-source release of 3,500 molecules of glutamate into the cleft directly underneath each synaptic vesicle, corresponding to ~100 mM glutamate in a 48 nm diameter synaptic vesicle. The diffusion coefficient of glutamate was 3e-6 cm^2^/s. The pre- and postsynaptic plasma membranes were set as reflective boundaries for glutamate molecules, which were allowed to diffuse out of the cleft. AMPA receptors were seeded into each synapse at a density of 1,500 receptors/μm^2^ and allowed to diffuse with a diffusion coefficient of 5e-10 cm^2^/s. AMPA receptors were constrained to remain within the PSD region of the postsynaptic membrane—defined as detailed above for analysis of ultrastructure—facing into the synaptic cleft. As a control, for a random configuration, receptors were seeded throughout the PSD region. For the clustered configuration, subregions of the postsynaptic membrane were defined that corresponded to the area of PSD clusters segmented from local density analysis (see above for segmentation details). 75% of the total AMPA receptors for each synapse were seeded into these cluster regions. The remaining 25% of receptors were seeded randomly outside of the cluster regions. After initial seeding, receptors were allowed to diffuse freely into and out of cluster regions during the simulation. The reaction scheme for glutamate binding to AMPA receptors and receptor activation used the same rate constants and states as in ref. 4. Briefly, receptors must bind two glutamate molecules before transitioning to a single open state. Each glutamate-bound state could also transition into a long-lived desensitized state. Simulations were run in steps of 1 μs for a total of 10 ms. For each vesicle and receptor configuration, 50 random seeds were used to initialize simulations, and the average of all 50 was taken as the response for that synaptic vesicle/receptor configuration.

### Statistics.

Statistical significance was set at *P < 0.05; **P < 0.01; ***P < 0.001. For nearest-neighbor distance analysis, significance was determined using a maximum absolute deviation (MAD) test. This is an envelope test that calculates the maximum absolute difference between the cumulative histogram of each trial (real data or simulated) versus the mean of all trials (data plus simulated). The p-value is calculated as the number of simulation trials with MAD values greater than that of the true data, divided by the total number of trials (90). For comparisons of the number of activated AMPA receptors in Monte-Carlo simulations of synaptic vesicle fusion, paired *t* tests were used. For determining the significance of the Spearman rank correlations between two parameters, two-sided paired permutation tests were performed, wherein one parameter was randomly permuted 10,000 times. All tests of statistical significance were performed in Python.

### Figure Preparation.

Figures were prepared in Adobe Illustrator. Graphs were generated using either Python or GraphPad Prism and imported into Adobe Illustrator for formatting. Two-dimensional images were imported from either IMOD, FIJI, or Napari (81, 86). Fig. 1*D* was processed using the Lamella in situ Clearing (LisC) algorithm to facilitate visualization (60). ChimeraX was used to visualize three-dimensional surfaces (85), with snapshots imported into Illustrator for figures. Simulation environments were rendered using Blender and exported as snapshots to Illustrator.

## Supplementary Material

Appendix 01 (PDF)

Dataset S01 (XLSX)

Movie S1. Example tomogram from Figure 2A, reconstructed at bin 8 (1.36 nm/pixel) and denoised with cryoCARE (4).

Movie S2. Movie of the segmentation shown in Figure 2B. Gray: plasma membranes; Orange: synaptic vesicles; Cyan: presynaptic protein density; Magenta: PSD protein density; Green: actin filaments.

Movie S3. Spin movie of the synaptic vesicle subtomogram shown in Figure 5A. The scale bar is 10 nm.

Movie S4. Spin movie of the synaptic vesicle subtomogram shown in Figure 5B. The atomic model of the C_1_-C_2_B-MUN-C_2_C fragment in the upright conformation is shown in red (PDB 7T7X). The scale bar is 10 nm.

Movie S5. Spin movie of the synaptic vesicle subtomogram shown in Figure 5C. The atomic model of the C_1_-C_2_B-MUN-C_2_C fragment in the lateral conformation is shown in red (PDB 7T7V). The SNARE/Syt1-C_2_B primary interface (PDB 5W5C) is also shown, red = syntaxin, green = SNAP25, blue = syntaptobrevin, yellow = complexin, gold = Syt1-C_2_B. The scale bar is 10 nm.

Movie S6. Spin movie of the synaptic vesicle subtomogram shown in Figure 5D. The atomic model of the SNARE/Syt1-C_2_B primary interface (PDB 5W5C) is shown, red = syntaxin, green = SNAP25, blue = syntaptobrevin, yellow = complexin, gold = Syt1-C_2_B. The scale bar is 10 nm.

Movie S7. Spin movie of the synaptic vesicle subtomogram shown in Figure 5E. The atomic model of the SNARE/Syt1-C_2_B primary interface (PDB 5W5C) is shown, red = syntaxin, green = SNAP25, blue = syntaptobrevin, yellow = complexin, gold = Syt1-C_2_B. The scale bar is 10 nm.

Movie S8. Movie of a single Monte Carlo reaction-diffusion simulation with AMPARs (blue receptors) placed in the random configuration throughout the PSD membrane. The locations of PSD nanoclusters are shown in magenta. A vesicle (orange) fuses and releases glutamate (yellow spheres) which diffuse rapidly and cause AMPAR opening (green receptors). Total time is 10 ms with simulation steps of 1 us.

Movie S9. Movie of a single Monte Carlo reaction-diffusion simulation run with AMPARs (blue receptors) placed in the clustered configuration throughout the PSD membrane. Coloring and placement of PSD nanoclusters, vesicles, and released glutamate are identical to video S8. Total time is 10 ms with simulation steps of 1 us.

## Data Availability

Statistical source data are available with the online version of the article. Unprocessed tomograms and Tabulated source data, ChimeraX (85) sessions, nanocluster coordinate files, and MCell/CellBlender simulations are available on the Stanford Digital Repository (https://doi.org/10.25740/xq706xv7995). All code for analysis is available through the Brunger Lab Github (https://github.com/brungerlab/synapse_tomograms). Unaligned motion-corrected tilt-series stacks and CryoCare (62) denoised, binned tomograms are available on EMPIAR (EMPIAR-12109) and EMDB (EMD-45161), respectively.

## References

[1] V. Braitenberg, A. Schüz, Cortex: Statistics and Geometry of Neuronal Connectivity (Springer Science & Business Media, 2013).

[2] J. M. Bekkers, C. F. Stevens, NMDA and non-NMDA receptors are co-localized at individual excitatory synapses in cultured rat hippocampus. Nature **341**, 230–233 (1989). 2571090 10.1038/341230a0

[3] A. K. McAllister, C. F. Stevens, Nonsaturation of AMPA and NMDA receptors at hippocampal synapses. Proc. Natl. Acad. Sci. U.S.A. **97**, 6173–6178 (2000). 10811899 10.1073/pnas.100126497 PMC18577

[4] J. Goncalves , Nanoscale co-organization and coactivation of AMPAR, NMDAR, and mGluR at excitatory synapses. Proc. Natl. Acad. Sci. U.S.A. **117**, 14503–14511 (2020). 32513712 10.1073/pnas.1922563117 PMC7321977

[5] D. Choquet, E. Hosy, AMPA receptor nanoscale dynamic organization and synaptic plasticities. Curr. Opin. Neurobiol. **63**, 137–145 (2020). 32416471 10.1016/j.conb.2020.04.003

[6] D. K. Patneau, M. L. Mayer, Structure-activity relationships for amino acid transmitter candidates acting at N-methyl-D-aspartate and quisqualate receptors. J. Neurosci. **10**, 2385–2399 (1990). 2165523 10.1523/JNEUROSCI.10-07-02385.1990 PMC6570388

[7] T. Ishikawa, Y. Sahara, T. Takahashi, A single packet of transmitter does not saturate postsynaptic glutamate receptors. Neuron **34**, 613–621 (2002). 12062044 10.1016/s0896-6273(02)00692-x

[8] S. Raghavachari, J. E. Lisman, Properties of quantal transmission at CA1 synapses. J. Neurophysiol. **92**, 2456–2467 (2004). 15115789 10.1152/jn.00258.2004

[9] D. Nair , Super-resolution imaging reveals that AMPA receptors inside synapses are dynamically organized in nanodomains regulated by PSD95. J. Neurosci. **33**, 13204–13224 (2013). 23926273 10.1523/JNEUROSCI.2381-12.2013 PMC6619720

[10] A.-H. Tang , A trans-synaptic nanocolumn aligns neurotransmitter release to receptors. Nature **536**, 210–214 (2016). 27462810 10.1038/nature19058 PMC5002394

[11] K. T. Haas , Pre-post synaptic alignment through neuroligin-1 tunes synaptic transmission efficiency. eLife **7**, e31755 (2018). 30044218 10.7554/eLife.31755 PMC6070337

[12] M. Sheng, E. Kim, The postsynaptic organization of synapses. Cold Spring Harb. Perspect. Biol. **3**, a005678 (2011). 22046028 10.1101/cshperspect.a005678 PMC3225953

[13] L. Chen , Stargazin regulates synaptic targeting of AMPA receptors by two distinct mechanisms. Nature **408**, 936–943 (2000). 11140673 10.1038/35050030

[14] X. Chen , PSD-95 family MAGUKs are essential for anchoring AMPA and NMDA receptor complexes at the postsynaptic density. Proc. Natl. Acad. Sci. U.S.A. **112**, E6983–E6992 (2015). 26604311 10.1073/pnas.1517045112 PMC4687590

[15] P. Opazo , CaMKII triggers the diffusional trapping of surface AMPARs through phosphorylation of stargazin. Neuron **67**, 239–252 (2010). 20670832 10.1016/j.neuron.2010.06.007

[16] R. B. Sutton, D. Fasshauer, R. Jahn, A. T. Brunger, Crystal structure of a SNARE complex involved in synaptic exocytosis at 2.4 A resolution. Nature **395**, 347–353 (1998). 9759724 10.1038/26412

[17] T. Weber , SNAREpins: Minimal machinery for membrane fusion. Cell **92**, 759–772 (1998). 9529252 10.1016/s0092-8674(00)81404-x

[18] N. Brose, A. G. Petrenko, T. C. Südhof, R. Jahn, Synaptotagmin: A calcium sensor on the synaptic vesicle surface. Science **256**, 1021–1025 (1992). 1589771 10.1126/science.1589771

[19] R. Fernández-Chacón , Synaptotagmin I functions as a calcium regulator of release probability. Nature **410**, 41–49 (2001). 11242035 10.1038/35065004

[20] H. T. McMahon, M. Missler, C. Li, T. C. Südhof, Complexins: Cytosolic proteins that regulate SNAP receptor function. Cell **83**, 111–119 (1995). 7553862 10.1016/0092-8674(95)90239-2

[21] Y. Lai , Complexin inhibits spontaneous release and synchronizes Ca2+-triggered synaptic vesicle fusion by distinct mechanisms. eLife **3**, e03756 (2014). 25122624 10.7554/eLife.03756 PMC4130161

[22] R. W. Cho , Genetic analysis of the Complexin trans-clamping model for cross-linking SNARE complexes in vivo. Proc. Natl. Acad. Sci. U.S.A. **111**, 10317–10322 (2014). 24982161 10.1073/pnas.1409311111 PMC4104896

[23] M. Xue , Binding of the complexin N terminus to the SNARE complex potentiates synaptic-vesicle fusogenicity. Nat. Struct. Mol. Biol. **17**, 568–575 (2010). 20400951 10.1038/nsmb.1791 PMC3172005

[24] A. Maximov, J. Tang, X. Yang, Z. P. Pang, T. C. Südhof, Complexin controls the force transfer from SNARE complexes to membranes in fusion. Science **323**, 516–521 (2009). 19164751 10.1126/science.1166505 PMC3235366

[25] T. C. Südhof, The presynaptic active zone. Neuron **75**, 11–25 (2012). 22794257 10.1016/j.neuron.2012.06.012 PMC3743085

[26] Y. Lai , Molecular mechanisms of synaptic vesicle priming by Munc13 and Munc18. Neuron **95**, 591–607.e10 (2017). 28772123 10.1016/j.neuron.2017.07.004 PMC5747255

[27] P. S. Kaeser , RIM proteins tether Ca2+ channels to Presynaptic active zones via a direct PDZ-domain interaction. Cell **144**, 282–295 (2011). 21241895 10.1016/j.cell.2010.12.029 PMC3063406

[28] R. Couteaux, M. Pécot-Dechavassine, Synaptic vesicles and pouches at the level of “active zones” of the neuromuscular junction. C R Acad Hebd Seances Acad. Sci. D **271**, 2346–2349 (1970). 4995202

[29] S. S. H. Wang , Fusion competent synaptic vesicles persist upon active zone disruption and loss of vesicle docking. Neuron **91**, 777–791 (2016). 27537483 10.1016/j.neuron.2016.07.005 PMC4991631

[30] C. Acuna, X. Liu, T. C. Südhof, How to make an active zone: Unexpected universal functional redundancy between RIMs and RIM-BPs. Neuron **91**, 792–807 (2016). 27537484 10.1016/j.neuron.2016.07.042

[31] I. Augustin, C. Rosenmund, T. C. Südhof, N. Brose, Munc13-1 is essential for fusion competence of glutamatergic synaptic vesicles. Nature **400**, 457–461 (1999). 10440375 10.1038/22768

[32] L. Deng, P. S. Kaeser, W. Xu, T. C. Südhof, RIM proteins activate vesicle priming by reversing autoinhibitory homodimerization of Munc13. Neuron **69**, 317–331 (2011). 21262469 10.1016/j.neuron.2011.01.005 PMC3063404

[33] C. Imig , The morphological and molecular nature of synaptic vesicle priming at presynaptic active zones. Neuron **84**, 416–431 (2014). 25374362 10.1016/j.neuron.2014.10.009

[34] C. Tan, S. S. H. Wang, G. de Nola, P. S. Kaeser, Rebuilding essential active zone functions within a synapse. Neuron **110**, 1498–1515.e8 (2022). 35176221 10.1016/j.neuron.2022.01.026 PMC9081183

[35] H. D. MacGillavry, Y. Song, S. Raghavachari, T. A. Blanpied, Nanoscale scaffolding domains within the postsynaptic density concentrate synaptic AMPA receptors. Neuron **78**, 615–622 (2013). 23719161 10.1016/j.neuron.2013.03.009 PMC3668352

[36] H. Sakamoto , Synaptic weight set by Munc13-1 supramolecular assemblies. Nat. Neurosci. **21**, 41–49 (2018). 29230050 10.1038/s41593-017-0041-9

[37] N. Rebola , Distinct nanoscale calcium channel and synaptic vesicle topographies contribute to the diversity of synaptic function. Neuron **104**, 693–710.e9 (2019). 31558350 10.1016/j.neuron.2019.08.014

[38] D. Maschi, V. A. Klyachko, Spatiotemporal regulation of synaptic vesicle fusion sites in central synapses. Neuron **94**, 65–73.e3 (2017). 28343869 10.1016/j.neuron.2017.03.006

[39] T. Biederer, P. S. Kaeser, T. A. Blanpied, Transcellular nanoalignment of synaptic function. Neuron **96**, 680–696 (2017). 29096080 10.1016/j.neuron.2017.10.006 PMC5777221

[40] M. Turk, W. Baumeister, The promise and the challenges of cryo-electron tomography. FEBS Lett. **594**, 3243–3261 (2020). 33020915 10.1002/1873-3468.13948

[41] Z. Wang , Structures from intact myofibrils reveal mechanism of thin filament regulation through nebulin. Science **375**, eabn1934 (2022). 35175800 10.1126/science.abn1934

[42] D. Tegunov, L. Xue, C. Dienemann, P. Cramer, J. Mahamid, Multi-particle cryo-EM refinement with M visualizes ribosome-antibiotic complex at 3.5 Å in cells. Nat. Methods **18**, 186–193 (2021). 33542511 10.1038/s41592-020-01054-7 PMC7611018

[43] R. Watanabe , The in situ structure of Parkinson’s disease-linked LRRK2. Cell **182**, 1508–1518.e16 (2020). 32783917 10.1016/j.cell.2020.08.004 PMC7869717

[44] R. Fernández-Busnadiego , Quantitative analysis of the native presynaptic cytomatrix by cryoelectron tomography. J. Cell Biol. **188**, 145–156 (2010). 20065095 10.1083/jcb.200908082 PMC2812849

[45] C. Papantoniou , Munc13- and SNAP25-dependent molecular bridges play a key role in synaptic vesicle priming. Sci. Adv. **9**, eadf6222 (2023). 37343100 10.1126/sciadv.adf6222 PMC10284560

[46] A. Martinez-Sanchez , Trans-synaptic assemblies link synaptic vesicles and neuroreceptors. Sci. Adv. **7**, eabe6204 (2021). 33674312 10.1126/sciadv.abe6204 PMC7935360

[47] R. Fernández-Busnadiego , Cryo-electron tomography reveals a critical role of RIM1α in synaptic vesicle tethering. J. Cell Biol. **201**, 725–740 (2013). 23712261 10.1083/jcb.201206063 PMC3664715

[48] Y.-T. Liu , Mesophasic organization of GABAA receptors in hippocampal inhibitory synapses. Nat. Neurosci. **23**, 1589–1596 (2020). 33139942 10.1038/s41593-020-00729-w PMC8048127

[49] C.-L. Tao , Differentiation and characterization of excitatory and inhibitory synapses by cryo-electron tomography and correlative microscopy. J. Neurosci. **38**, 1493–1510 (2018). 29311144 10.1523/JNEUROSCI.1548-17.2017 PMC5815350

[50] R. Danev, B. Buijsse, M. Khoshouei, J. M. Plitzko, W. Baumeister, Volta potential phase plate for in-focus phase contrast transmission electron microscopy. Proc. Natl. Acad. Sci. U.S.A. **111**, 15635–15640 (2014). 25331897 10.1073/pnas.1418377111 PMC4226124

[51] M. Nulati Yesibolati , Electron inelastic mean free path in water. Nanoscale **12**, 20649–20657 (2020). 32614016 10.1039/d0nr04352d

[52] W. J. Rice , Routine determination of ice thickness for cryo-EM grids. J. Struct. Biol. **204**, 38–44 (2018). 29981485 10.1016/j.jsb.2018.06.007 PMC6119488

[53] R. G. Held , Synapse and active zone assembly in the absence of presynaptic Ca2+ channels and Ca2+ entry. Neuron **107**, 667–683.e9 (2020). 32616470 10.1016/j.neuron.2020.05.032 PMC7442750

[54] K. M. Harris, F. E. Jensen, B. Tsao, Three-dimensional structure of dendritic spines and synapses in rat hippocampus (CA1) at postnatal day 15 and adult ages: Implications for the maturation of synaptic physiology and long-term potentiation. J. Neurosci. **12**, 2685–2705 (1992). 1613552 10.1523/JNEUROSCI.12-07-02685.1992 PMC6575840

[55] J. L. Dickerson, P.-H. Lu, D. Hristov, R. E. Dunin-Borkowski, C. J. Russo, Imaging biological macromolecules in thick specimens: The role of inelastic scattering in cryoEM. Ultramicroscopy **237**, 113510 (2022). 35367900 10.1016/j.ultramic.2022.113510 PMC9355893

[56] M. J. Peet, R. Henderson, C. J. Russo, The energy dependence of contrast and damage in electron cryomicroscopy of biological molecules. Ultramicroscopy **203**, 125–131 (2019). 30773415 10.1016/j.ultramic.2019.02.007 PMC6495108

[57] A. Rigort, J. M. Plitzko, Cryo-focused-ion-beam applications in structural biology. Arch. Biochem. Biophys. **581**, 122–130 (2015). 25703192 10.1016/j.abb.2015.02.009

[58] M. Schaffer , Optimized cryo-focused ion beam sample preparation aimed at in situ structural studies of membrane proteins. J. Struct. Biol. **197**, 73–82 (2017). 27444390 10.1016/j.jsb.2016.07.010

[59] A. Rigort , Focused ion beam micromachining of eukaryotic cells for cryoelectron tomography. Proc. Natl. Acad. Sci. U.S.A. **109**, 4449–4454 (2012). 22392984 10.1073/pnas.1201333109 PMC3311327

[60] F. J. B. Bäuerlein , Cryo-electron tomography of large biological specimens vitrified by plunge freezing. bioRxiv [Preprint] (2023). https://www.biorxiv.org/content/10.1101/2021.04.14.437159v3 (Accessed 3 February 2024).

[61] J. J. Peters , A feature-guided, focused 3D signal permutation method for subtomogram averaging. J. Struct. Biol. **214**, 107851 (2022). 35346811 10.1016/j.jsb.2022.107851 PMC9149098

[62] T.-O. Buchholz, M. Jordan, G. Pigino, F. Jug, Cryo-CARE: Content-aware image restoration for cryo-transmission electron microscopy data. arXiv [Preprint] (2018). 10.48550/arXiv.1810.05420 (Accessed 1 May 2020). 31326025

[63] D. Tegunov, P. Cramer, Real-time cryo-electron microscopy data preprocessing with Warp. Nat. Methods **16**, 1146–1152 (2019). 31591575 10.1038/s41592-019-0580-y PMC6858868

[64] K. Grushin, R. V. Kalyana Sundaram, C. V. Sindelar, J. E. Rothman, Munc13 structural transitions and oligomers that may choreograph successive stages in vesicle priming for neurotransmitter release. Proc. Natl. Acad. Sci. U.S.A. **119**, e2121259119 (2022). 35135883 10.1073/pnas.2121259119 PMC8851502

[65] Q. Zhou , The primed SNARE-complexin-synaptotagmin complex for neuronal exocytosis. Nature **548**, 420–425 (2017). 28813412 10.1038/nature23484 PMC5757840

[66] Q. Zhou , Architecture of the synaptotagmin-SNARE machinery for neuronal exocytosis. Nature **525**, 62–67 (2015). 26280336 10.1038/nature14975 PMC4607316

[67] A. M. Getz , High-resolution imaging and manipulation of endogenous AMPA receptor surface mobility during synaptic plasticity and learning. Sci. Adv. **8**, eabm5298 (2022). 35895810 10.1126/sciadv.abm5298 PMC9328687

[68] T. C. Südhof, Neurotransmitter release: The last millisecond in the life of a synaptic vesicle. Neuron **80**, 675–690 (2013). 24183019 10.1016/j.neuron.2013.10.022 PMC3866025

[69] S. Li , Asynchronous release sites align with NMDA receptors in mouse hippocampal synapses. Nat. Commun. **12**, 677 (2021). 33514725 10.1038/s41467-021-21004-x PMC7846561

[70] G. Malagon, J. Myeong, V. A. Klyachko, Two forms of asynchronous release with distinctive spatiotemporal dynamics in central synapses. eLife **12**, e84041 (2023). 37166282 10.7554/eLife.84041 PMC10174687

[71] C. S. Wang, N. L. Chanaday, L. M. Monteggia, E. T. Kavalali, Probing the segregation of evoked and spontaneous neurotransmission via photobleaching and recovery of a fluorescent glutamate sensor. eLife **11**, e76008 (2022). 35420542 10.7554/eLife.76008 PMC9129874

[72] M. Armstrong , Microscale fluid behavior during cryo-EM sample blotting. Biophys. J. **118**, 708–719 (2020). 31952802 10.1016/j.bpj.2019.12.017 PMC7004840

[73] B. Quade , Membrane bridging by Munc13-1 is crucial for neurotransmitter release. eLife **8**, e42806 (2019). 30816091 10.7554/eLife.42806 PMC6407922

[74] E. Eggermann, I. Bucurenciu, S. P. Goswami, P. Jonas, Nanodomain coupling between Ca^2+^ channels and sensors of exocytosis at fast mammalian synapses. Nat. Rev. Neurosci. **13**, 7–21 (2011). 22183436 10.1038/nrn3125 PMC3617475

[75] A. T. Brunger, J. Leitz, The core complex of the Ca2+-triggered presynaptic fusion machinery. J. Mol. Biol. **435**, 167853 (2023). 36243149 10.1016/j.jmb.2022.167853 PMC10578080

[76] P. Jonas, G. Major, B. Sakmann, Quantal components of unitary EPSCs at the mossy fibre synapse on CA3 pyramidal cells of rat hippocampus. J. Physiol. **472**, 615–663 (1993). 7908327 10.1113/jphysiol.1993.sp019965 PMC1160505

[77] J. M. Bekkers, G. B. Richerson, C. F. Stevens, Origin of variability in quantal size in cultured hippocampal neurons and hippocampal slices. Proc. Natl. Acad. Sci. U.S.A. **87**, 5359–5362 (1990). 2371276 10.1073/pnas.87.14.5359 PMC54323

[78] L. Forti, M. Bossi, A. Bergamaschi, A. Villa, A. Malgaroli, Loose-patch recordings of single quanta at individual hippocampal synapses. Nature **388**, 874–878 (1997). 9278048 10.1038/42251

[79] A. Maximov, Z. P. Pang, D. G. R. Tervo, T. C. Südhof, Monitoring synaptic transmission in primary neuronal cultures using local extracellular stimulation. J. Neurosci. Methods **161**, 75–87 (2007). 17118459 10.1016/j.jneumeth.2006.10.009

[80] W. J. H. Hagen, W. Wan, J. A. G. Briggs, Implementation of a cryo-electron tomography tilt-scheme optimized for high resolution subtomogram averaging. J. Struct. Biol. **197**, 191–198 (2017). 27313000 10.1016/j.jsb.2016.06.007 PMC5287356

[81] J. R. Kremer, D. N. Mastronarde, J. R. McIntosh, Computer visualization of three-dimensional image data using IMOD. J. Struct. Biol. **116**, 71–76 (1996). 8742726 10.1006/jsbi.1996.0013

[82] A. Martinez-Sanchez, I. Garcia, S. Asano, V. Lucic, J. J. Fernandez, Robust membrane detection based on tensor voting for electron tomography. J. Struct. Biol. **186**, 49–61 (2014). 24625523 10.1016/j.jsb.2014.02.015

[83] M. Chen , Convolutional neural networks for automated annotation of cellular cryo-electron tomograms. Nat. Methods **14**, 983–985 (2017). 28846087 10.1038/nmeth.4405 PMC5623144

[84] A. Rigort , Automated segmentation of electron tomograms for a quantitative description of actin filament networks. J. Struct. Biol. **177**, 135–144 (2012). 21907807 10.1016/j.jsb.2011.08.012

[85] T. D. Goddard , UCSF ChimeraX: Meeting modern challenges in visualization and analysis. Protein Sci. **27**, 14–25 (2018). 28710774 10.1002/pro.3235 PMC5734306

[86] C.-L. Chiu, N. Clack, T. N. Community, napari: A python multi-dimensional image viewer platform for the research community. Microsc. Microanal. **28**, 1576–1577 (2022).

[87] J. R. Stiles, D. Van Helden, T. M. Bartol, E. E. Salpeter, M. M. Salpeter, Miniature endplate current rise times <100 μ s from improved dual recordings can be modeled with passive acetylcholine diffusion from a synaptic vesicle. Proc. Natl. Acad. Sci. U.S.A. **93**, 5747–5752 (1996). 8650164 10.1073/pnas.93.12.5747 PMC39132

[88] J. R. Stiles, T. M. Bartol, “Monte Carlo methods for simulating realistic synaptic microphysiology using MCell” in Computational Neuroscience: Realistic Modeling for Experimentalists, E. De Schutter, Ed. (CRC Press, Boca Raton, FL, 2001), pp. 87–127.

[89] R. A. Kerr , Fast Monte Carlo simulation methods for biological reaction-diffusion systems in solution and on surfaces. SIAM J. Sci. Comput. **30**, 3126–3149 (2008). 20151023 10.1137/070692017 PMC2819163

[90] A. Baddeley , On tests of spatial pattern based on simulation envelopes. Ecol. Monogr. **84**, 477–489 (2014).

