# Supplementary material for: Nanoscale architecture of synaptic vesicles and scaffolding complexes revealed by cryo-electron tomography
Source: Proc Natl Acad Sci U S A. 2024 Jun 26;121(27):e2403136121. doi: 10.1073/pnas.2403136121 (PMC11228483; doi:10.1073/pnas.2403136121)
Supplement: Supplementary file 1 — Appendix 01 (PDF) [file pnas.2403136121.sapp.pdf]

PNAS

Supporting Information for

Nanoscale architecture of synaptic vesicles and scaffolding complexes revealed by cryo-electron tomography

Richard G. Held, Jiahao Liang, and Axel T. Brunger

*Corresponding author: Axel Brunger
Email: brunger@stanford.edu

This PDF file includes:

Supporting text
Figures S1 to S5
Legends for Movies S1 to S9
Legends for Datasets S1
SI References

Other supporting materials for this manuscript include the following:

Movies S1 to S9
Datasets S1

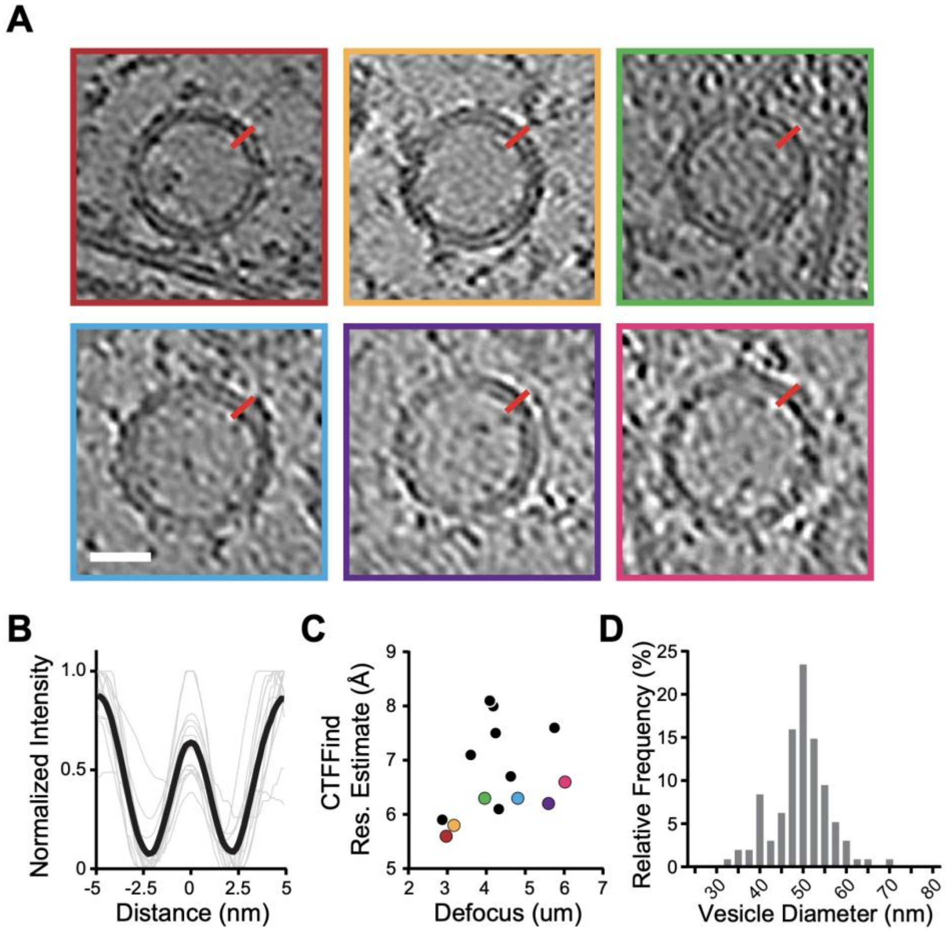

Fig. S1. Approximation of Tomogram Resolution. A) Example XY tomogram slices of synaptic vesicles from denoised tomograms in the dataset. Red lines indicate the position of line scans across the vesicle membrane. Scale bar = 20 nm, pixel size = 1.36 nm/pixel. **B)** Normalized line scans through vesicle membranes, as shown in A. Values from individual tomograms are shown in gray, and the average is in black (N = 14 synapses). **C)** Relationship between tomogram defocus and the maximum fit resolution reported by CTFFIND for the 0-degree tilt projection image for all tomograms in the dataset. Points are color-coded to match the example images in A (N = 14 synapses). **D)** Frequency distribution of vesicle diameters for membrane-proximal synaptic vesicles (N = 93 vesicles, 14 synapses).

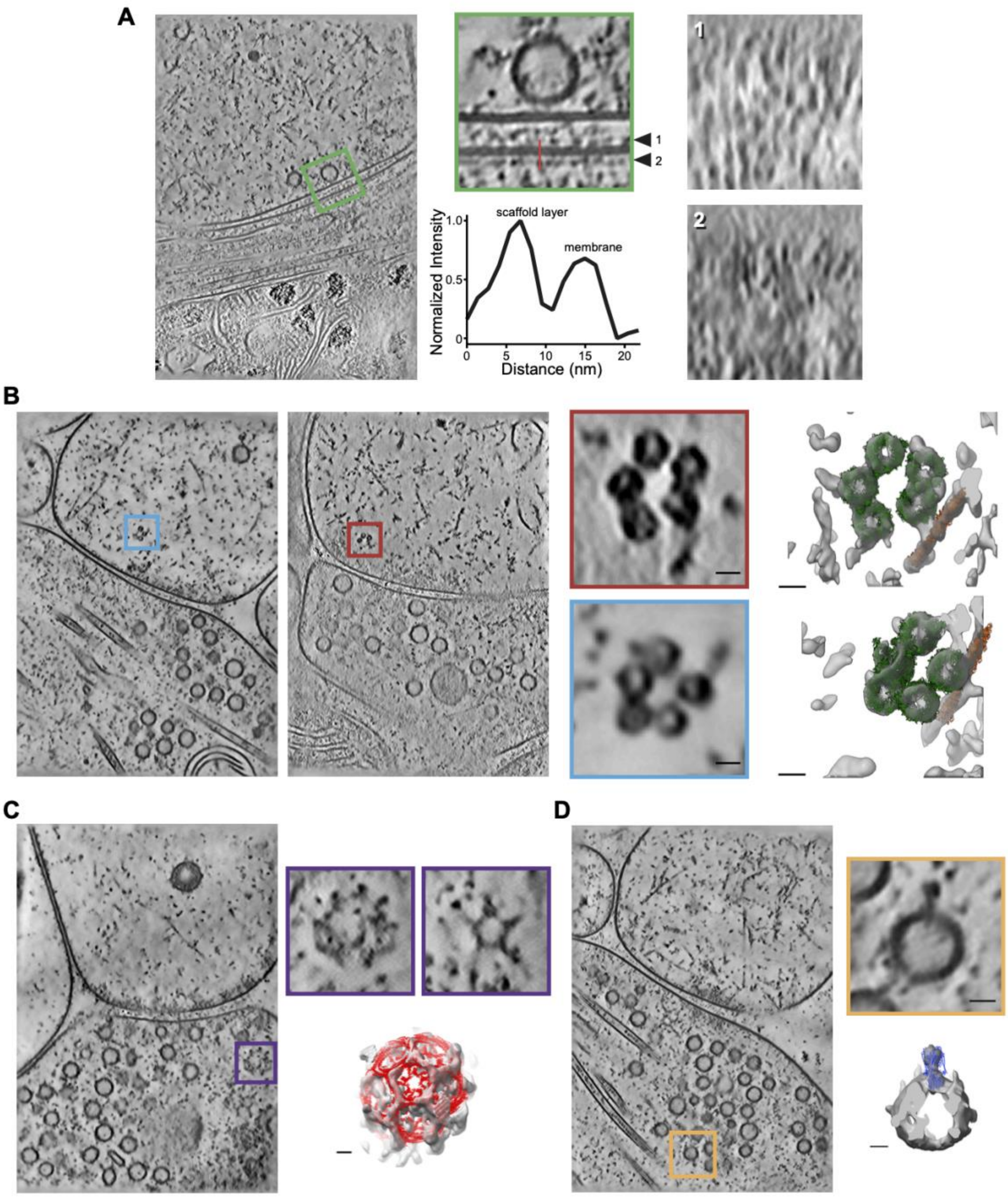

Fig. S2. **Additional Examples of Notable Synaptic Features. A)** An example of a likely inhibitory synapse. An XY slice of a tomogram, denoised and missing wedge corrected by Isonet (1), is shown on the left. The green boxed region shows a membrane-proximal synaptic vesicle. On the postsynaptic side of the cleft a band-like region of protein density can be seen lining the intracellular surface of the plasma membrane, consistent with reports of gephyrin-expressing inhibitory synapses (2). Line scans (top middle panel, red line) show this density layer to be ~10 nm wide. XZ slices (right) on either side of the postsynaptic plasma membrane show the putative receptor layer (1) and the

intracellular gephyrin layer (2). **B)** Isonet corrected XY slices of tomograms with supramolecular rings of TriC in the postsynapse. Blue and red boxed regions were visualized using the IMOD slicer tool. Extracted volumes (far right) could accommodate structures of TRIC (PDB 5GW5, shown in green), forming a ring-like super-structure in apparent contact with postsynaptic actin filaments (PDB 6BNO, shown in orange). **C)** An Isonet corrected XY slice from a tomogram with an apparently empty presynaptic clathrin basket boxed in purple. Two XY slices of the basket are shown on the right at different Z-planes. An extracted volume (bottom right) could accommodate a D6 clathrin cage (PDB 1XI4, shown in red). **D)** An Isonet corrected XY slice from a tomogram showing a vesicle with a putative vATPase. The orange-boxed region was visualized using the IMOD slicer tool (top right) (3). Extracted volumes (bottom right) could accommodate the structure of rat brain v-ATPase (PDB 6VQ6, shown in blue). All scale bars are 10 nm.

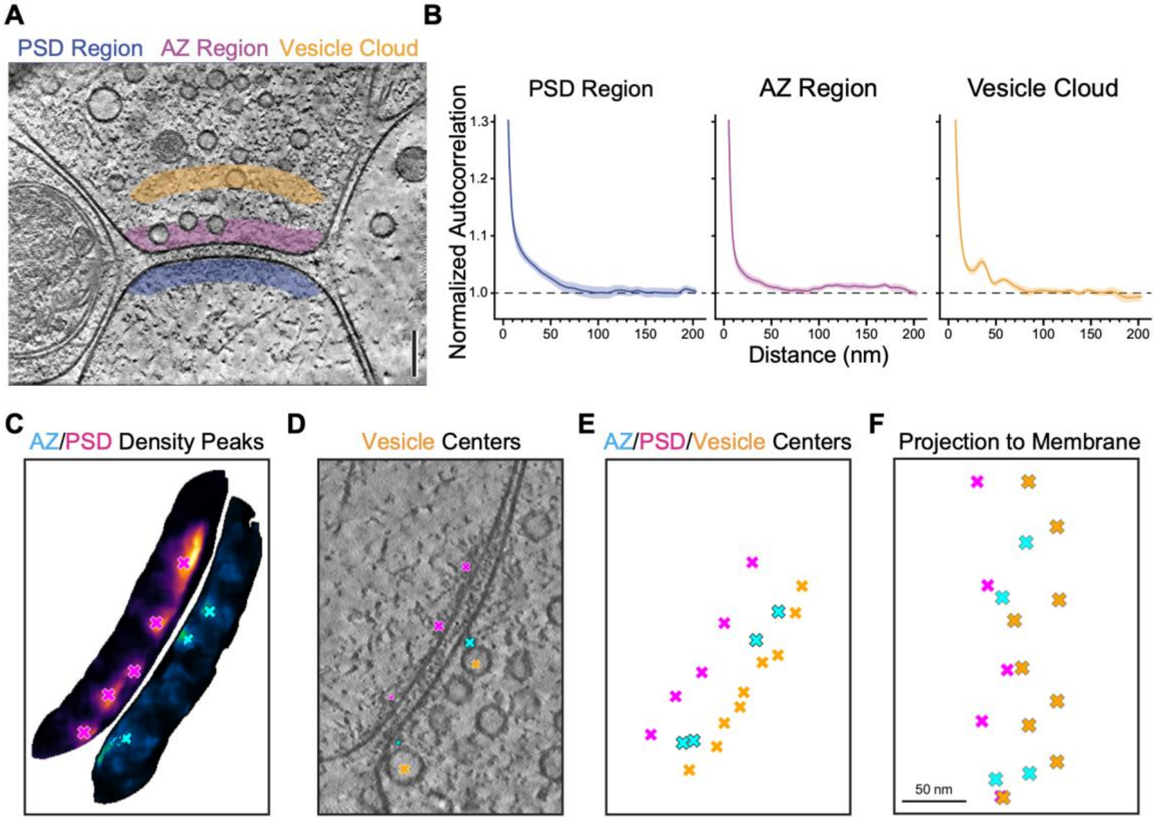

Fig. S3. **Autocorrelation Analysis and Nanocluster Localization. A)** An XY slice through a Wiener-deconvolved tomogram. Colored regions indicate the slices through masked volumes used for autocorrelation analysis. Blue is the PSD region, pink is the active zone region (excluding vesicle volume), and orange is the same volume as the PSD region but shifted into the vesicle cloud. Scale bar = 100 nm. **B)** Normalized rotationally averaged autocorrelation plots of the masked volumes indicated in A. Plots show average plus standard error for all synapses in the dataset (N = 13 synapses). Dashed lines indicate the expected value of the same volume with voxels shuffled within the mask. **C)** Maximum intensity projections of AZ and PSD local density maps in the XY plane. Color-coded (Cyan = AZ, Magenta = PSD) 'X' markers indicate the center of mass of each cluster. **D)** An XY slice through the same tomogram showing the center of mass of membrane-proximal vesicles in orange. Unmarked vesicles have centers in different planes of the tomogram volume. **E)** XY projections of the centers of AZ and PSD clusters and vesicle centers. **F)** The same center positions, as shown in C, were rotated and projected onto the plane of the postsynaptic plasma membrane.

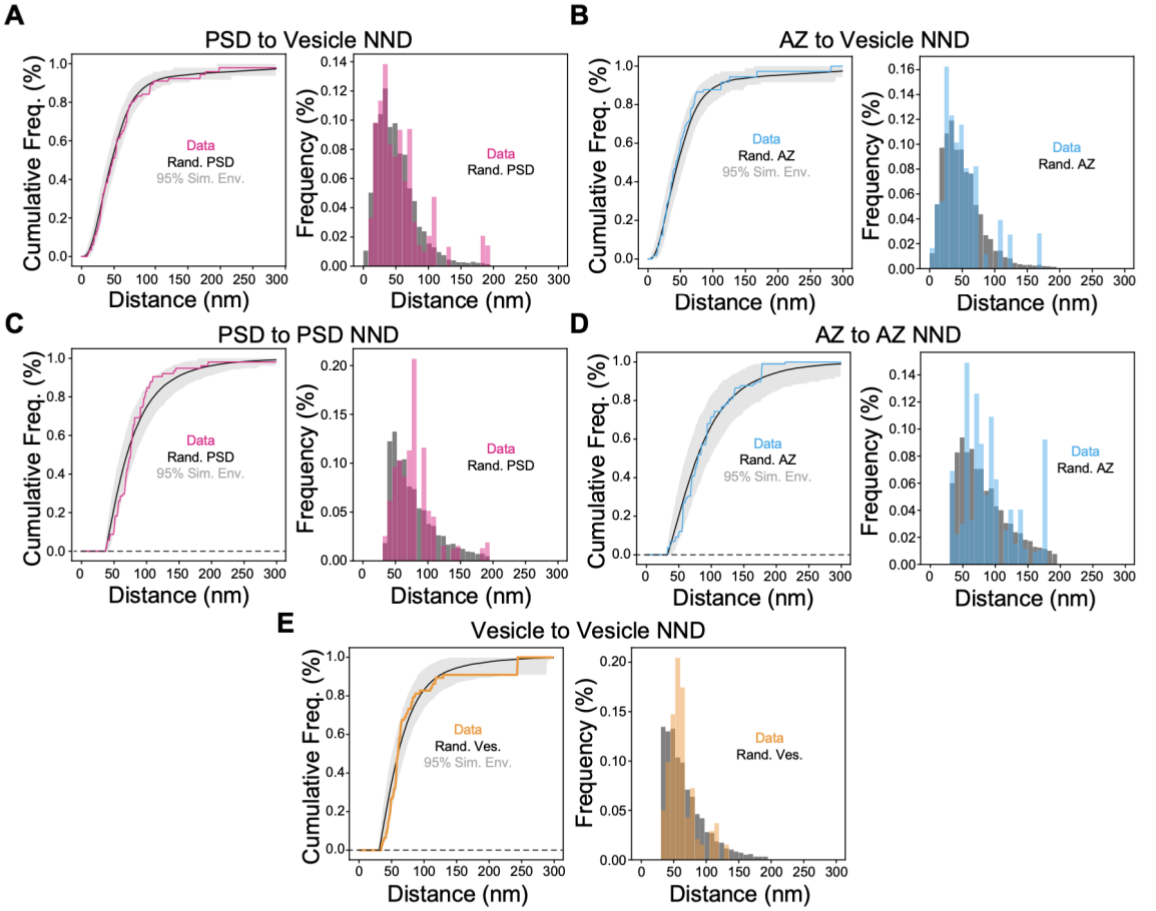

Fig. S4. Additional Quantification of Scaffold and Vesicle Clustering and Trans-Synaptic Alignment. A) Cumulative frequency (left) and frequency (right) histograms of the lateral distance between PSD clusters and their nearest neighbor membrane-proximal vesicle (NND). The distribution of the observed distance data is shown in magenta compared to simulation means in black. The 95% simulation envelope of 1000 simulated random PSD cluster positions is shown in gray (N= 65 PSD clusters, 12 synapses). **B)** Cumulative frequency (left) and frequency (right) histograms of the lateral distance between AZ clusters and their nearest neighbor membrane-proximal vesicle. Observed distance data are shown in cyan, and simulations in black/gray as in A (N= 58 AZ clusters, 12 synapses). **C)** Cumulative frequency (left) and frequency (right) histograms of the nearest-neighbor distance between PSD nanoclusters. Measured data are shown in magenta, and simulations in black/gray as in A (N= 68 PSD clusters, 13 synapses). **D)** Cumulative frequency (left) and frequency (right) histograms of the nearest-neighbor distance between AZ nanoclusters. Observed distance data are shown in magenta, and simulations in black/gray as in A (N= 61 AZ clusters, 13 synapses). **E)** Cumulative frequency (left) and frequency (right) histograms of the nearest-neighbor distance between membrane-proximal vesicles. Observed distance data are shown in magenta, and simulations in black/gray as in A (N= 85 vesicles, 12 synapses).

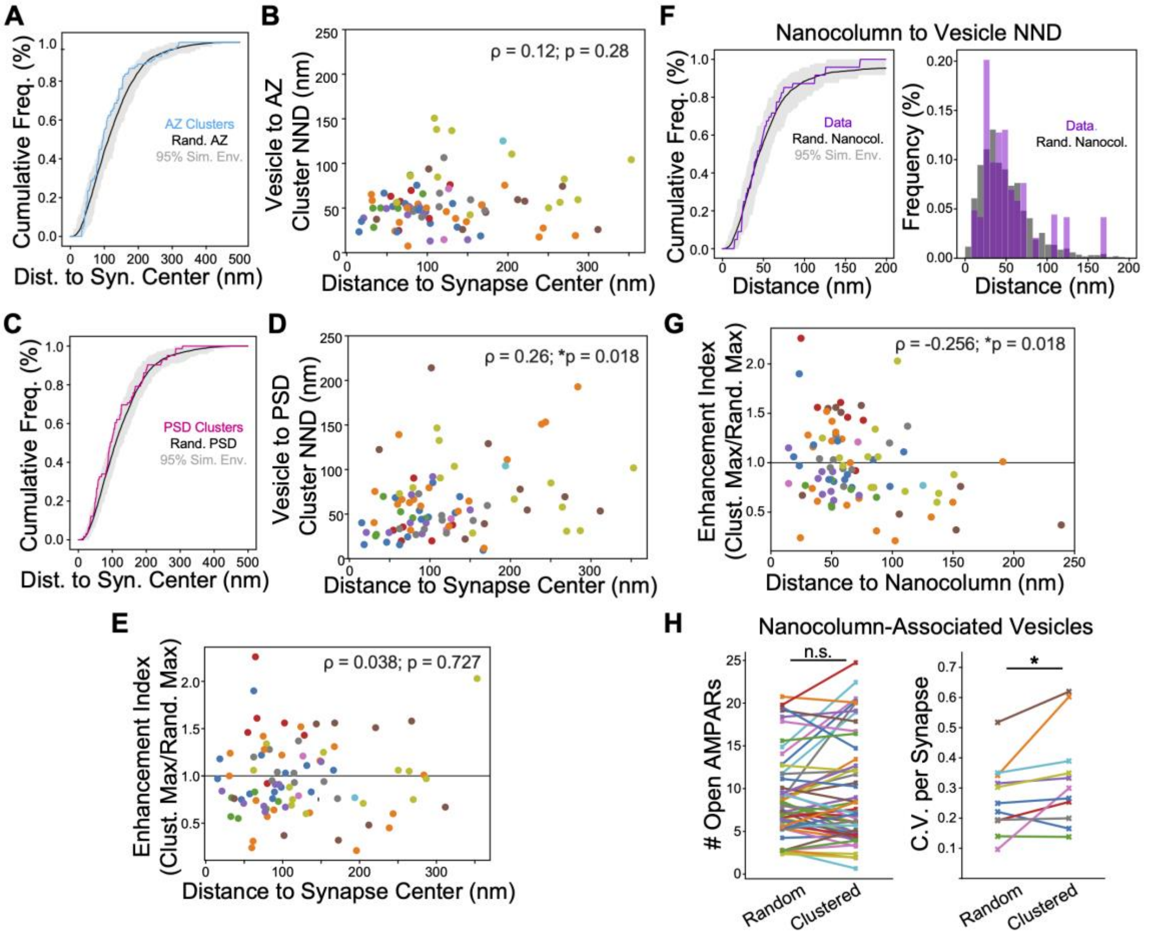

Fig. S5. Impact of Vesicle Distance to Synapse Center and Nanocolumn Alignment. **A)** Cumulative histogram of the distance between an AZ nanocluster and the center of the synapse (N = 58 AZ nanoclusters, 12 synapses). **B)** Scatter plot of the distance between each vesicle and the center of the synapse versus the distance to the nearest-neighbor AZ nanocluster (N = 85 vesicles, 12 synapses). **C)** Cumulative histogram of the distance between a PSD nanocluster and the center of the synapse (N = 68 PSD nanoclusters, 12 synapses). **D)** Scatter plot of the distance between each vesicle and the center of the synapse versus the distance to the nearest-neighbor PSD nanocluster (N = 85 vesicles, 12 synapses, *p = 0.018). **E)** Scatter plot of the distance between each vesicle and the center of the synapse versus the simulated enhancement index (N = 85 vesicles, 12 synapses). **F)** Cumulative frequency (left) and frequency (right) histograms of the nearest-neighbor distance between vesicles and nanocolumns (N= 51 nanocolumns, 12 synapses).
G) Scatter plot of the distance between each vesicle and the nearest nanocolumn versus the simulated enhancement index (N = 85 vesicles, 12 synapses, *p = 0.018). **H)** The maximum number of open AMPA receptors in response to single vesicle fusion events of nanocolumn associated vesicles with receptors in random versus clustered configurations (left). Coefficient of variation (C.V.) values for each synapse (right). *p = 0.048 as determined by paired t-test.

Video S1 (separate file). Example tomogram from Figure 2A, reconstructed at bin 8 (1.36 nm/pixel) and denoised with cryoCARE (4).

Video S2 (separate file). Movie of the segmentation shown in Figure 2B. Gray: plasma membranes; Orange: synaptic vesicles; Cyan: presynaptic protein density; Magenta: PSD protein density; Green: actin filaments.

Video S3 (separate file). Spin movie of the synaptic vesicle subtomogram shown in Figure 5A. The scale bar is 10 nm.

Video S4 (separate file). Spin movie of the synaptic vesicle subtomogram shown in Figure 5B. The atomic model of the C_1-C_2B-MUN-C_2C fragment in the upright conformation is shown in red (PDB 7T7X). The scale bar is 10 nm.

Video S5 (separate file). Spin movie of the synaptic vesicle subtomogram shown in Figure 5C. The atomic model of the C_1-C_2B-MUN-C_2C fragment in the lateral conformation is shown in red (PDB 7T7V). The SNARE/Syt1-C_2B primary interface (PDB 5W5C) is also shown, red = syntaxin, green = SNAP25, blue = syntaptobrevin, yellow = complexin, gold = Syt1-C_2B. The scale bar is 10 nm.

Video S6 (separate file). Spin movie of the synaptic vesicle subtomogram shown in Figure 5D. The atomic model of the SNARE/Syt1-C_2B primary interface (PDB 5W5C) is shown, red = syntaxin, green = SNAP25, blue = syntaptobrevin, yellow = complexin, gold = Syt1-C_2B. The scale bar is 10 nm.

Video S7 (separate file). Spin movie of the synaptic vesicle subtomogram shown in Figure 5E. The atomic model of the SNARE/Syt1-C_2B primary interface (PDB 5W5C) is shown, red = syntaxin, green = SNAP25, blue = syntaptobrevin, yellow = complexin, gold = Syt1-C_2B. The scale bar is 10 nm.

Video S8 (separate file). Movie of a single Monte Carlo reaction-diffusion simulation with AMPARs (blue receptors) placed in the random configuration throughout the PSD membrane. The locations of PSD nanoclusters are shown in magenta. A vesicle (orange) fuses and releases glutamate (yellow spheres) which diffuse rapidly and cause AMPAR opening (green receptors). Total time is 10 ms with simulation steps of 1 us.

Video S9 (separate file). Movie of a single Monte Carlo reaction-diffusion simulation run with AMPARs (blue receptors) placed in the clustered configuration throughout the PSD membrane. Coloring and placement of PSD nanoclusters, vesicles, and released glutamate are identical to video S8. Total time is 10 ms with simulation steps of 1 us.

Dataset S1 (separate file). Microsoft Excel document with three tabs: **1)** ultrastructure data displayed in Figure 2; **2)** A vesicle dataframe listing the tomogram, coordinates, distance to AZ membrane, AZ nanocluster nearest-neighbor distance, PSD nanocluster nearest-neighbor distance, vesicle nearest-neighbor distance, Monte Carlo reaction diffusion simulation maximum number of open AMPARs for the clustered and random configurations, and the enhancement index, distance to the PSD membrane center-of-mass, and distance to a AZ/PSD nanocolumn for every vesicle in the dataset. **3)** A PSD nanocluster dataframe listing the tomogram, coordinates, AZ nanocluster nearest-neighbor distance, vesicle nearest-neighbor distance, and PSD nanocluster nearest-neighbor distance for every PSD nanocluster in the dataset. **4)** An AZ nanocluster dataframe listing the tomogram, coordinates, PSD nanocluster nearest-neighbor distance, vesicle nearest-neighbor distance, and AZ nanocluster nearest-neighbor distance for every PSD nanocluster in the dataset.

SI References

1. Y.-T. Liu, *et al.*, Isotropic reconstruction for electron tomography with deep learning. *Nat Commun* **13**, 6482 (2022).
2. C.-L. Tao, *et al.*, Differentiation and Characterization of Excitatory and Inhibitory Synapses by Cryo-electron Tomography and Correlative Microscopy. *J Neurosci* **38**, 1493–1510 (2018).
3. J. R. Kremer, D. N. Mastronarde, J. R. McIntosh, Computer Visualization of Three-Dimensional Image Data Using IMOD. *Journal of Structural Biology* **116**, 71–76 (1996).
4. T.-O. Buchholz, M. Jordan, G. Pigino, F. Jug, Cryo-CARE: Content-Aware Image Restoration for Cryo-Transmission Electron Microscopy Data. *arXiv:1810.05420 [cs]* (2018) (September 28, 2021).
